# Identification of an Amylomaltase from the Halophilic Archaeon *Haloquadratum walsbyi* by Functional Metagenomics: Structural and Functional Insights

**DOI:** 10.3390/life12010085

**Published:** 2022-01-07

**Authors:** Claudia Leoni, Caterina Manzari, Hai Tran, Peter N. Golyshin, Graziano Pesole, Mariateresa Volpicella, Luigi R. Ceci

**Affiliations:** 1 Institute of Biomembranes, Bioenergetics and Molecular Biotechnologies, Consiglio Nazionale delle Ricerche, 70126 Bari, Italy; c.leoni@ibiom.cnr.it (C.L.); graziano.pesole@uniba.it (G.P.); 2 Department of Biosciences, Biotechnologies and Biopharmaceutics, University of Bari Aldo Moro, 70126 Bari, Italy; c.manzari@ibiom.cnr.it; 3 Centre for Environmental Biotechnology, School of Natural Sciences, Bangor University, Bangor LL57 2UW, UK; t.hai@bangor.ac.uk (H.T.); p.golyshin@bangor.ac.uk (P.N.G.)

**Keywords:** amylomaltase, 4-α-glucanotransferase, halophile, *Archaea*, *Haloquadratum walsbyi*, saltern, functional metagenomics

## Abstract

Amylomaltases are prokaryotic 4-α-glucanotransferases of the GH77 family. Thanks to the ability to modify starch, they constitute a group of enzymes of great interest for biotechnological applications. In this work we report the identification, by means of a functional metagenomics screening of the crystallization waters of the saltern of Margherita di Savoia (Italy), of an amylomaltase gene from the halophilic archaeon *Haloquadratum walsbyi*, and its expression in *Escherichia coli* cells. Sequence analysis indicated that the gene has specific insertions yet unknown in homologous genes in prokaryotes, and present only in amylomaltase genes identified in the genomes of other *H. walsbyi* strains. The gene is not part of any operon involved in the metabolism of maltooligosaccharides or glycogen, as it has been found in bacteria, making it impossible currently to assign a precise role to the encoded enzyme. Sequence analysis of the *H. walsbyi* amylomaltase and 3D modelling showed a common structure with homologous enzymes characterized in mesophilic and thermophilic bacteria. The recombinant *H. walsbyi* enzyme showed starch transglycosylation activity over a wide range of NaCl concentrations, with maltotriose as the best acceptor substrate compared to other maltooligosaccharides. This is the first study of an amylomaltase from a halophilic microorganism.

## 1. Introduction

Recombinant enzymes have been extensively studied for their use in biocatalysis processes in the food, pharmaceutical, chemical, biofuels, and textile industries. The first approaches involved the use of mesophilic enzymes; however, these have shown poor stability at high temperatures or at extreme pH values [1]. In recent years, biotechnological studies have therefore focused on enzymes obtained from extremophilic microorganisms, called extremozymes, which have high stability in extreme conditions of temperature or pH, in the presence of organic solvents and at high ionic concentrations. The use of extremozymes is remarkably advantageous as it allows chemical syntheses to be carried out without employing polluting organic solvents and high energy inputs, resulting in reductions in environmental damage and economic costs, as well as in the disposal of toxic substances [1,2].

Most of the extremozymes currently studied for possible applications in industrial processes belong to the class of hydrolases (glycoside hydrolases, proteases, lipases and esterases [1,2,3,4,5,6]). Among them, enzymes capable of modifying starch have aroused great interest due to the considerable abundance of this carbohydrate in nature and its widespread use in food and industrial applications [7,8,9]. Starch is synthesized in plants as a heterogeneous compound, mainly containing the two polymers amylose and amylopectin. Amylose is essentially a linear α-1,4-polyglucoside, containing up to 1000 glucose units. Amylopectin consists of α-1,4-polyglucoside molecules of 10–60 glucose units, containing α-1,6-branches of the α-1,4-polyglucosides of 15–45 glucose units. The side chains are further branched, giving rise to amylopectin molecules containing on average two million glucose units. Typically, native starches consist of a mixture of 15–30% amylose and 70–85% amylopectin. However, the size of the two polymers and their ratio are different from plant to plant [7]. After solubilization in hot water, starches have properties not yet compatible with food and industrial applications, which can be obtained by subsequent enzymatic modifications. High-temperature active enzymes are therefore particularly appropriate for starch modifications [10,11,12]. However, extremozymes active at low temperatures or high salt concentrations are also being studied for possible applications on starch [13,14].

Starch-modifying enzymes include: (i) glycoside hydrolases (α-amylase, β-amylase, glucoamylase, α-glucosidase), which hydrolyze the α-1,4 glycosidic bonds; (ii) de-branching enzymes (isoamylase and pullulanases), which hydrolyze the α-1,6 glycosidic bonds; (iii) transferases (cyclodextrin glycosyltransferase, amylomaltase, branching enzyme), which, after the hydrolysis of the α-1,4 glycosidic bonds of the donor molecules, transfer the glucoside with the newly formed reducing end to the 4′-position of a α-1,4′ glucan acceptor [15]. Most of these starch-modifying enzymes are classified in distinct glycoside hydrolase (GH) families in the CAZy (Carbohydrate-Active enZymes) database (https://www.cazy.org/, accessed on 30 November 2021) [16].

In recent years, several studies have been conducted on the specific group of transferases known as amylomaltases. They are enzymes of the CAZy GH77 family, which contains specific 4-α-glucanotransferases identified only in plants and algae (known as disproportionating or D-enzymes) and in prokaryotes (amylomaltases). Most of the GH77 enzymes reported in CAZy are of bacterial origin (13,240 in November 2021), while only 83 are from *Archaea* and 117 from plants and algae. While in plants D-enzymes are essential for starch metabolism [17], in bacteria amylomaltases are associated with different functions, such as bacterial growth on maltooligosaccharides and degradation of glycogen. The amylomaltase coding gene (*malQ*) can in fact be both present in the maltose-inducible *malPQ* operon and associated with the *glgBXCAP* operon, identified in many groups of bacteria for glycogen synthesis and degradation [18]. No studies have been carried out for establishing the physiological role of amylomaltases in *Archaea*.

Studies on the 3D structures of proteins of the GH77 family are relatively few. For plants, structures have been determined for *Arabidopsis thaliana* [19] and potato [20]. For bacteria, structures have been obtained for amylomaltases from *Aquifex aeolicus* (Protein Data Bank, PDB, accession number 1TZ7), *Corynebacterium glutamicum* [21], *E. coli* [22], *Streptococcus agalactiae* [23], *Thermus brockianus* [24], *T. thermophilus* AT-62 (formerly indicated as *T. aquaticus* [25]) [26]. No structural studies are available for amylomaltases from *Archaea*. Structurally, GH77 enzymes contain an α-amylase-type (β/α)_8_ barrel catalytic domain (named Subdomain A), carrying specific insertions between its strands, indicated as Subdomains B1, B2 and B3. Przylas et al. [26] provided a detailed description of Subdomains B1–B3 of the *T. thermophilus* amylomaltase. The three subdomains form a continuous ring around the C-terminal edge of the (β/α)_8_ barrel and bind to the large amylose substrates. The catalytic residues Asp293, Glu340 and Asp395 are located in Subdomain A. The active site also contains other conserved amino acids, such as Tyr59, Asp213, Arg291 and His394, which contribute toward binding the substrate molecules and constitute, together with the catalytic residues, the core of the catalytic cleft. An extended and flexible loop (residues 247–255) partially covers the active-site cleft, with possible roles in substrate binding and product dissociation. This region, known as the 250s loop, is also conserved throughout the GH77 family [26].

Furthermore, based on sequence and structural evidence, amylomaltases can be classified in at least four distinct groups [27]: (i) amylomaltases related to the *T. thermophilus* AT-62 sequence; (ii) amylomaltases related to enzymes identified in the genus *Borrellia*, characterized by the presence of specific amino acids in otherwise highly conserved positions. In fact, this group contains several subgroups which differ in some functionally important amino acids, such as lysine instead of the highly conserved Arg291 (hereafter the amylomaltase numbering will be according to the *T. thermophilus* amylomaltase sequence [26]), as in the case of the amylomaltases from *B. burgdorferi* and *B. duttonii*, or glutamic acid instead of the catalytic transition-state stabilizer D395 in the enzymes from *B. duttonii* and *B. turicatae*. (iii) amylomaltase related to the plant enzyme DPE2 (disproportionating enzymes type 2), which contains an insertion of about 140 amino acids in the region between the amino acids of the catalytic triad D293 and E340 and two copies of the starch-binding domain (SBD) of the carbohydrate-binding module (CBM) of family 20 in the N-terminal domain preceding Subdomain A [28]. (iv) amylomaltases with a longer N-terminus, related to the *E. coli* enzyme. Moreover, for this group of enzymes, bioinformatics and docking analysis allowed for hypothesizing the presence of a new SBD within the extended N-terminal domain [29].

Amylomaltases have aroused interest for biotechnological applications, as they have been found useful for the production of (i) slow digestible starch, a modified starch with a reduced susceptibility to enzymatic hydrolyses, useful for the production of foods with a low glycemic index; (ii) cycloamyloses, circular α-1,4-glucans containing up to a few hundred glucose units, with possible applications as carrier in the food, pharmaceutical and chemical industries; (iii) thermoreversible starch gels, as substitute for animal gelatin in the food industry (see [25] and references therein). Even if several studies on amylomaltases from thermophilic and hyper-thermophilic bacteria and *Archaea* have been carried out, amylomaltases from halophiles have never been studied. In this study we report the identification, by means of a functional metagenomics screening of the saltern of Margherita di Savoia (Italy), of the gene for an amylomaltase from the halophilic archaeon *H. walsbyi* and the expression of the enzyme in *E. coli* cells. We also report the first insights on its functional activity and structural characteristics.

## 2. Materials and Methods

### 2.1. Sampling and Purification of eDNA

Water samples were collected from the crystallization pond “Imperatrice” (36% of salinity, 30 °C, pH 7.20) of the Margherita di Savoia saltern, located on the south-eastern coast of Italy (Lat. 41°23′10” N, Long. 16°7′14” E) on June 2017. Water sampling and eDNA extraction were conducted as described by Leoni et al. [30].

### 2.2. Generation and Screening of a Fosmid Library

A fosmid library was generated from the Imperatrice pond eDNA using the pCC1FOS expression vector and titrated according to the manufacturer’s protocol (CopyControl™ Fosmid Library Production Kit, Epicentre Cat. No CCFOS110). Functional screening for cellulase, glycoside hydrolase and esterase/lipase activities was carried out on an LB/agar medium supplemented with chloramphenicol (12.5 μg/mL) and suitable substrates (carboxymethyl cellulose, X-Gal and tributyrin, respectively), as already described in Placido et al. [31]. Fosmid DNA extraction from selected clones and sequencing was performed as already reported [31].

### 2.3. Bioinformatic Analysis

Bioinformatic analysis of fosmid insert sequences was carried out using the A-GAME (A GAlaxy suite for functional MEtagenomics) pipeline [32], a web service implemented within the Galaxy platform [33] allowing for incorporation of widely used bioinformatic tools for the analysis of eDNA sequence data. In particular, a workflow was developed for the analysis of fosmid inserts, made by the following programs: Bowtie-2 [34], Trimmomatic [35], Flash [36] and FastQ interlacer (present in the Galaxy tool shed: https://toolshed.g2.bx.psu.edu/, accessed on 30 November 2018) for sequence pre-processing; Meta-velvet [37] and Sanger End Attacher (present in the Galaxy tool shed: https://toolshed.g2.bx.psu.edu/, accessed on 30 November 2018) for sequence assembly; MetageneMark [38] for gene prediction and PFAM annotator [39] for functional annotation.

Alignments of the *H. walsbyi* amylomaltase sequence with those of the GH77 groups or with archaeal homologs were carried out by Clustal Omega [40]. Phylogenetic trees were generated using the MEGA11 software [41], applying the maximum-likelihood method and the Jones–Taylor–Thornton model for amino acid substitution. Bootstrap support values were estimated using 500 pseudo-replicates.

Structural modelling of the *H. walsbyi* amylomaltase was performed using the Phyre2 server [42].

### 2.4. Amplification and Cloning of the Amylomaltase Gene

A total of 52 ng of fosmid DNA was used as the template for amylomaltase gene amplification. PCR was performed in presence of 10 pmoles of the primers:

18B-FP: 5′-TTGTATTTCCAGGGC/ATGCAGTTTGATCGACAGG-3′ and

18B-RP: 5′-CAAGCTTCGTCA/TCAGTCACGGACATGTTCGAGTG-3′,

Corresponding to the initial and terminal sequences of the *H. walsbyi* amylomaltase gene, respectively (the underlined sequences correspond to vector regions), 10 mM dNTP and 1 U of Triple Master Polymerase mix (Eppendorf, Hamburg, Germany) combined in a total of 50 μL of the specific High-Fidelity Buffer supplied by the enzyme vendor (Eppendorf). The amplification reaction was conducted in a thermal cycler according to the following program: 5 min at 94 °C and 35 cycles of 30 s at 94 °C, 30 s at the annealing temperature of 65 °C and 1 min at 72 °C. After purification, 1 μL of the amplification product was inserted in the *BseRI*-linearized p15TV-L expression vector (Addgene, Watertown, MA, USA) using the In-Fusion™ PCR Cloning Kit (Takara Bio, Shiga, Japan) procedure.

### 2.5. Expression and Purification of Hw-A Recombinant Protein

The recombinant vector containing the amylomaltase gene was cloned in *E. coli* Origami (DE3) cells (Invitrogen, Waltham, MA, USA) through a heat shock procedure. The transformed cells were spread on plates containing LB agar medium supplemented with 25 μg/μL ampicillin and incubated at 37 °C overnight. A single colony of recombinant cells was picked and grown in 500 mL LB broth supplemented with 50 μg ampicillin at 37 °C, until the culture optical density at 600 nm (OD600) reached 0.5. Then the expression of the recombinant protein was induced by adding iso-propyl-1-thio-β-D-galactopyranoside (IPTG) (1 mM final concentration) and by incubating the cell culture at 20 °C with shaking overnight. After induction, the cells were centrifuged for 5 min at 13,000 rpm and resuspended in 10 mL of binding buffer (1 M NaCl, 20 mM sodium phosphate pH 7 and 10 mM imidazole). The cells were lysed using the Vibra-Cell Sonicator (Sonics, Newtown, CT, USA) for 2.5 min with alternating on/off cycles of 15 s each at 50% amplitude. The lysate was centrifuged at 18,500× *g* for 30 min and the supernatant was analyzed by SDS-PAGE. The expression of the recombinant protein was verified through a Western blot assay using the Mouse Anti Penta Histidine Tag: HRP antibody (Bio-Rad, Hercules, CA, USA). The protein was purified from the lysate supernatant by affinity chromatography on a HisTrap-HP column (GE Healthcare, Chicago, IL, USA) by a step-wise procedure using elution buffers (1 M NaCl, 20 mM sodium phosphate, pH 7) containing increasing concentrations of imidazole (10 mM, 50 mM, 100 mM, 500 mM). Chromatography fractions were analyzed through SDS/PAGE. Fractions containing the recombinant protein were dialyzed in 20 mM sodium phosphate buffer and concentrated using 30 kDa Amicon columns (Pro Affinity Concentration Kit Ni-NTA, Amicon, Seattle, WA, USA). The protein concentration was determined using the Bradford assay kit (Bio-Rad, Hercules, CA, USA).

### 2.6. Amylomaltase Transglycosylation and Hydrolytic Activity Assays

Transglycosylation activity of amylomaltase was determined by incubating different amounts (0.01–10 μg range) of enzyme with 0.15% starch, as donor substrate, and either 0.05% of glucose (G8270, Sigma-Aldrich, Milan, Italy), maltose (M5885, Sigma-Aldrich, Milan, Italy), maltotriose (M8378, Sigma-Aldrich, Milan, Italy), Maltotetraose (sc–218667C, Santa Cruz Biotechnology, Heidelberg, Germany), maltohexaose (M9153, Sigma-Aldrich, Milan, Italy) and maltoheptaose (M7753, Sigma-Aldrich, Milan, Italy) as acceptor substrates, in 30 μL of 50 mM sodium phosphate buffer pH 7 supplemented with 1 M NaCl. The reaction mix was incubated for 1 h at 40 °C. The assay was also performed at different temperatures (30–70 °C range), pH values (5.2–8 range), and NaCl concentrations (0–4 M range). The starch degradation was determined by the iodine solution method described by Mehboob et al. [43].

The thermal stability assay was carried out by incubating 2.5 μg of enzyme in 50 mM sodium phosphate buffer pH 7, 1 M NaCl for 30 min at different temperatures (40–70 °C) before the transglycosylation assay with starch and maltotriose as substrates.

The hydrolytic activity was measured incubating 2.5 μg of protein with 0.15% starch solution in the same buffer as the previous assay, at 40 °C for 1 h. The production of glucose was measured using the Glucose Liquid Color reagent (Human Diagnostic Worldwide, Magdeburg, Germany), as described by Mehboob et al. [43]. For both assays, positive control reactions were carried out using amylase from *Bacillus licheniformis* (A3306, Sigma-Aldrich, Milan, Italy). The reactions were also carried out without enzymes, as negative control.

### 2.7. Thermal Shift

Thermal shift assays were carried out by incubation of 2.5 μg enzyme with 5 μL of 200 X SYPROTM Orange protein gel stain (Thermo Fisher Scientific, Waltham, MA, USA) in 50 μL of 50 mM sodium phosphate buffer supplemented with 1 M NaCl. The reaction was conducted in the real-time PCR system (QuantStudio7Flex Real-Time PCR, Applied Biosystems, Life Technologies, Waltham, MA, USA) and melting curves were obtained at 5 °C increasing steps in the 25–95 °C range of temperature. The increase in temperature was set up at 0.5 °C/s. The assays were performed with different NaCl concentrations (0–4 M range), pH values (5.2–8 range) and DMSO concentrations (0–20% range).

## 3. Results and Discussion

### 3.1. Fosmid Library Generation, Screening and Identification of an Amylomaltase Coding Sequence

One liter of the water from the Imperatrice pond of the Margherita di Savoia saltern, with a salinity of 36%, was sampled and filtered through Stericup filters (0.22 μM) for prokaryote isolation. The microbiota DNA extracted from the filters was fragmented in 30–40 Kb segments and cloned in the pCC1-FOS vector to generate a fosmid library. The library titer was estimated at 2.1 × 10^6^ cfu/mL. The screening of about 10,000 clones, carried out using LB agar plates (20 × 20 cm) containing one of the substrates tributyrin, carboxyl-methylcellulose or X-gal, allowed for the identification of positive clones for the activities of cellulases (clones hydrolyzing carboxyl-methylcellulose), β-glycosyl-hydrolases (clones hydrolyzing X-Gal) and carboxylesterases/lipases (clones hydrolyzing tributyrin). From selected clones, fifteen fosmid inserts were fully sequenced and annotated using the A-GAME tool [32]. Analysis of the DNA sequences obtained (about 500 kb) allowed for the identification of 590 possible coding sequences, 306 of which could be annotated into PFAM families (data not shown). Among the identified protein coding sequences, the gene for a putative amylomaltase was identified (GenBank accession n. MZ422727).

BLAST analysis of the gene sequence showed the highest percentages of identity (about 82%) with homologous genes annotated in the genomes of the *H. walsbyi* strains C23 (GenBank accession n. FR746099.1) and DSM16790 (GenBank accession n. AM180088.1), cultivated from cells isolated in Australia (Cheetham Salt Works, Geelong, Victoria) [44] and Spain (saltern of Brac del Port, Alicante) [45], respectively. The encoded protein showed an identity percentage of about 99% with a 4-α-glucanotransferase from the *H. walsbyi* J07HQW1 strain, identified in the course of a deep metagenomic sequencing of the hypersaline Lake Tyrrell (Victoria, Australia) [46]. The analysis of the sequenced fosmid showed that the amylomaltase gene (*malQ*) is not located in any possible operon related to metabolism of oligosaccharides or glycogen, as described for homologous bacterial genes [18]. It is located 1685 nucleotides downstream of the gene coding for an RNA-guided endonuclease of the TnpB family and 380 nucleotides upstream of the gene coding for an hypothetical protein, followed by the gene coding for a DNA-glycosylase (data not shown). Homologous genes are also present in the sequenced *H. walsbyi* genomes, FR746099.1 and AM180088.1. The *malQ* gene is actively transcribed under both light and dark conditions, albeit below the average level of gene expression [47].

Amylomaltases are gaining interest in the food and pharmaceutical industries for their ability to modify starch, and several enzymes from thermophilic and hyper-thermophilic prokaryotes have already been produced as recombinant molecules and characterized [25]. Since amylomaltases from halophilic microorganisms have never been isolated, we considered it interesting to characterize the putative *H. walsbyi* enzyme.

**Figure 1:**
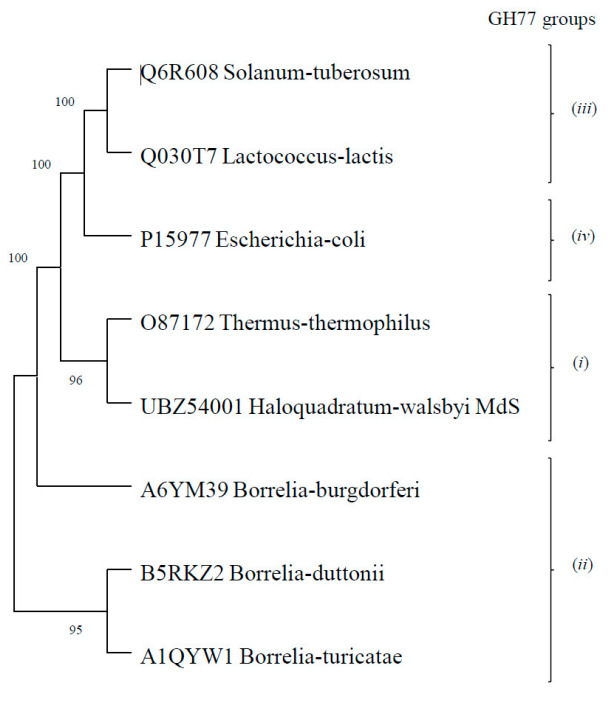
Phylogenetic tree of Hw-A and representative sequences of the four GH77 groups. The sequences used for the phylogenetic analysis are indicated by their UniProt accession number (GenBank for Hw-A) and by the name of the microorganism of origin. Phylogenetic analysis was carried out by the MEGA11 software suite [41]. Bootstrap values are reported close to nodes. The Clustal alignment of the eight sequences, used as input file for the MEGA analysis, is reported in the Supplementary Material (Figure S1). GH77 groups refer to amylomaltases related to: (i) the *T. thermophilus* enzyme; (ii) the enzymes identified in the *Borrellia* genus; (iii) the potato enzyme DPE2; (iv) the *E. coli* enzyme (see Introduction and [27] for details).

Sequence analysis of the *H. walsbyi* amylomaltase (hereafter indicated as Hw-A) showed the presence of peculiar insertion sequences not present in other amylomaltases (Figure S1), raising the question whether it can be assigned to one of the four groups in which the enzymes have been distinguished [27]. Figure 1 reports the phylogenetic analysis of Hw-A and representative sequences of the four GH77 groups.

**Figure 2:**
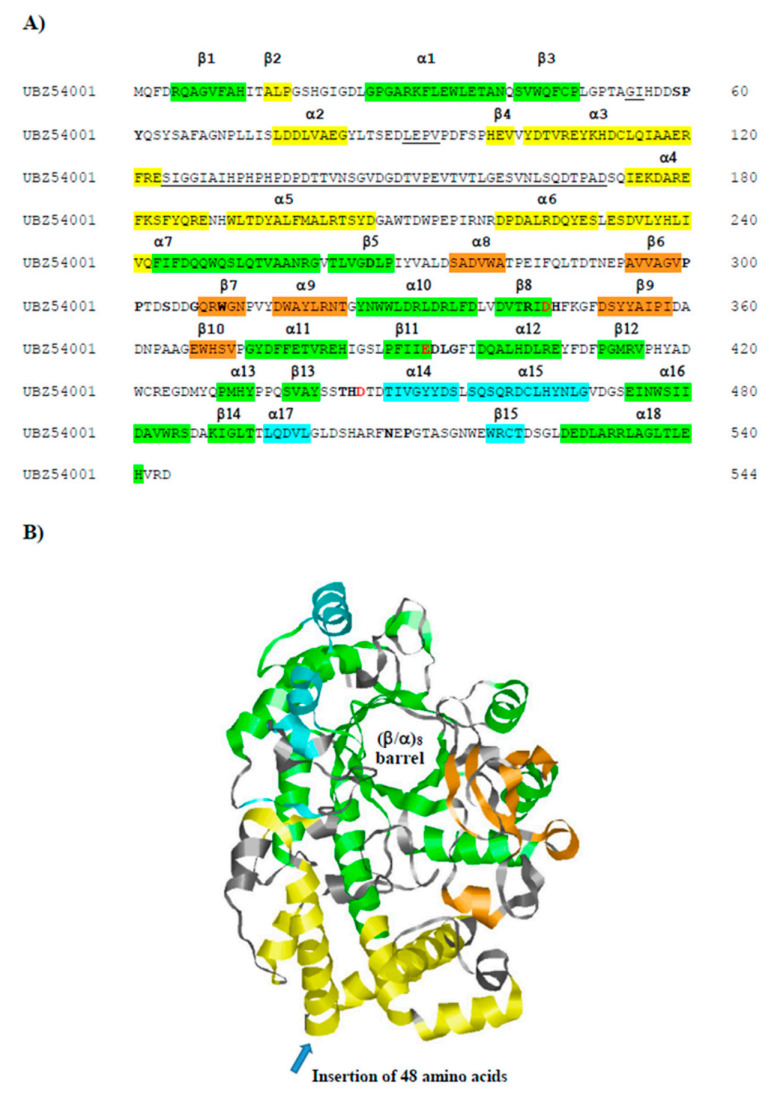
(**A**) Amino acid sequence of the *H. walsbyi* amylomaltase Hw-A. Structural elements are according to the homologous enzyme from *T. thermophilus* [26]. Green regions refer to the (β/α)_8_ barrel (Subdomain A); the other colored regions correspond to Subdomain B1 (orange), Subdomain B2 (yellow) and Subdomain B3 (cyan). The helix α7 is composed by sequences belonging to B1 and B2 subdomains. The insertion regions not present in the *T. thermophilus* enzyme are underlined. The three catalytic residues are reported in red bold, while the other conserved amino acids in the active site and 250 s loop regions are shown in bold. (**B**) A 3D model of Hw-A, as obtained by the Phyre2 modeling program [42] on the basis of the *T. thermophilus* amylomaltase structure (PDB, 1ESW). Structural elements have the same colors reported in the sequence in Panel A. The blue arrow indicates the insertion point of the largest extra sequence not present in the reference structure.

Hw-A is closer to the *T. thermophilus* sequence (group *i*), but it also shows some peculiar insertions. The longest insertion of 48 amino acids is between the α3 and α4 helices of Subdomain B2 (Figure 2A). Despite the large insertion, a well-conserved 3D structure was predicted for Hw-A by the Phyre2 modelling program [42], with 100% confidence for the *T. thermophilus* amylomaltase structure (PDB, 1ESW) (Figure 2B). The insertion of the 48 amino acids is located on the opposite side of the catalytic site of the amylomaltases (Figure 2B). In addition, sequence comparison allowed us to confirm the presence of structurally and functionally important amino acids described in the *T. thermophilus* homolog [26] (Figure 2A): in particular, for the region of the active site, the three amino acids of the catalytic triad D293, E340 and D395, four additional amino acids required for substrate orientation and reaction specificity (Y59, D213, R291, H394), six amino acids forming part of the cleft around the active center (S57, P58, D341, G343, T393, P466) and other conserved amino acids (W258, H294, L342, N464). Amino acids were less conserved in the 250 s loop. Only the amino acids P247, P248, S252 and G255 of the nine loop residues were maintained. Table S1 shows the corresponding numbering of the above-mentioned amino acids in the amylomaltase sequences of *T. thermophilus* and *H. walsbyi*. BLAST analysis (not shown) indicated that the Hw-A sequence was more conserved among halophilic *Archaea* (with percentages of identity in the 56–99% range for the first 50 hits) than among bacteria, for which the percentages of identity were not higher than 40%. Furthermore, a Clustal analysis of Hw-A with the 83 archaeal amylomaltases present in GH77 showed that the insertion of 48 amino acids was present only in the available *H. walsbyi* sequences (Figure S2). This result indicates a specific insertion event limited so far to *H. walsbyi* among *Archaea*. BLASTP analysis of the 48-amino-acid insert did not provide significant results. The *H. walsbyi* enzyme was also characterized by a high percentage of acidic amino acids (16.7%), the highest value detected so far among extreme amylomaltases [25]. This reflects the origin of the enzyme from a halophilic archaeal microorganism. Halophilic *Archaea*, indeed, are characterized by possessing of enzymes with increased surface charges that are required to adapt to the high intracellular salt concentration [48].

### 3.2. Expression of the Recombinant Hw-A Enzyme

**Figure 3:**
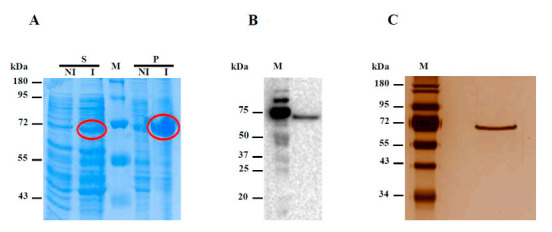
Expression of the recombinant Hw-A protein. (**A**) SDS-PAGE of the proteins expressed by transformed *E. coli* cells. The possible recombinant Hw-A protein was circled in red. S: supernatant; P: pellet; NI: not induced; I: induced; M: Page Ruler Prestained Protein Ladder (Thermo Scientific, Waltham, MA, USA). (**B**) Western blot analysis of protein contained in the supernatant of *E. coli* sonicated cells, using an anti-His antibody. M: Precision Plus Standard Protein Western C marker (Bio-Rad). (**C**) SDS-PAGE of the purified Hw-A protein, stained with silver staining.

The amylomaltase Hw-A gene was cloned in the p-15TV-L expression vector and the recombinant protein was expressed using the *E. coli* Origami DE3 cell system. The expressed protein contains the Hw-A protein and 21 additional amino acids (including a six His-tag at its N-terminus) encoded by the p15TV-L vector, resulting in a final molecular weight of about 67 kDa. The protein, as shown by the electrophoretic analysis, was generally present mostly in the cell pellet obtained after sonication, while smaller amounts were present in the supernatant. Different expression experiments allowed us to establish that an increase of the soluble protein fraction was possible by inducing the expression with 1 mM IPTG at 20 °C (Figure 3A), which was then used as the routine expression procedure. The expression was confirmed through Western blot assay using an anti-His-tag antibody (Figure 3B).

The recombinant protein was purified by Ni-affinity chromatography using a discontinuous 10–500 mM imidazole gradient. The protein was mostly present in the fractions obtained using 100 mM imidazole (Figure S3), which were collected, dialyzed against 50 mM sodium phosphate buffer and concentrated through the use of Amicon columns with a 30 kDa cutoff (Figure 3C). Typically, the protein yield was 2 mg per L of *E. coli* culture, as evaluated by the Bradford assays.

### 3.3. Transglycosylation Activity

**Figure 4:**
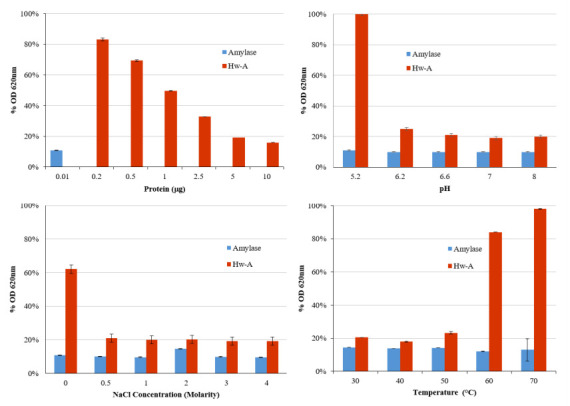
Hw-A starch transglycosylation activity assays. Different protein amounts, pH, NaCl concentrations and temperatures were assayed. The ordinate axis shows the percentage reduction values of absorbance at OD 620 nm of the starch–iodine complex compared to reactions carried out without enzyme.

Amylomaltases are able to carry out a starch transglycosylation reaction, with the transfer of the glucan carrying the newly formed reducing end to an acceptor substrate, such as a maltooligosaccharide [49,50]. Accordingly, the activity of Hw-A was monitored by measuring the decrease in the absorbance of the starch–iodine complex at 620 nm, according to Mehboob et al. [43]. The reactions were carried out for 1 h with different enzyme amounts and under different conditions of pH, NaCl concentrations and temperature, in the presence of maltotriose as acceptor substrate. A total of 10 ng of α-amylase from *B. licheniformis* was used as positive control in each assay (Figure 4). The results obtained indicated the optimal conditions to be used in the subsequent activity assays: 2.5 µg of protein, 1 M NaCl, pH 7 at 40 °C.

**Figure 5:**
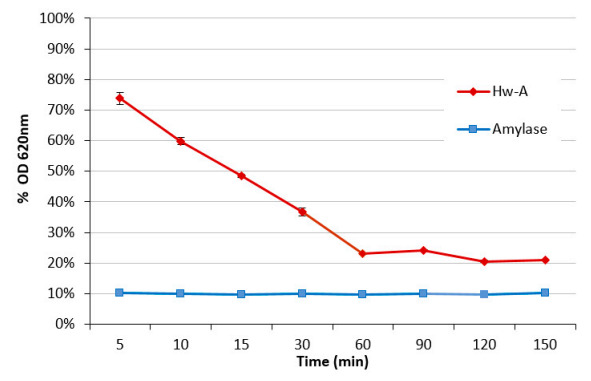
Hw-A starch transglycosylation activity assay at different times. The ordinate axis shows the starch–iodine complex percentage reduction absorbance at OD 620 nm, compared to the blank reaction.

A time-course experiment was then carried out (Figure 5) showing that a saturation condition is reached after 120 min of incubation with a starch residue of 20%. A similar result was also observed for the amylomaltase from *Pyrobacculum calidifontis* [43].

**Figure 6:**
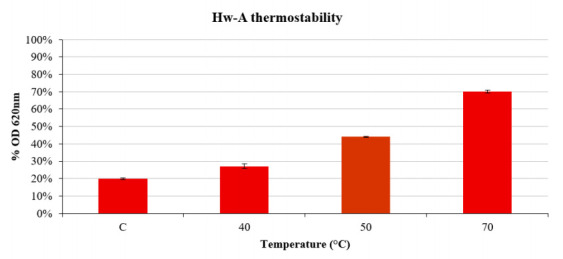
Hw-A starch transglycosylation activity assay with the enzyme pretreated at different temperatures. The ordinate axis shows the percentage reduction values of absorbance at OD 620 nm of the starch–iodine complex compared to reactions carried out without the enzyme. The pretreatment temperature is indicated on the abscissa axis. C stands for the control reaction without any pretreatment.

The thermal stability of the protein was assayed by heating 2.5 μg of enzyme at different temperatures for 30 min before the starch transglycosylation assay, carried out under optimal conditions using maltotriose as acceptor substrate. The activity of the protein was affected by the heating pretreatment (Figure 6).

**Figure 7:**
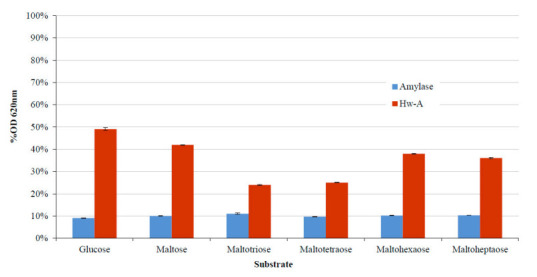
Starch transglycosylation assays with different acceptor substrates. The percentage reduction values of absorbance at OD 620 nm of the starch–iodine complex are reported with different acceptor substrates.

Further transglycosylation assays were performed under optimal conditions to identify the best acceptors among the maltooligosaccharides glucose, maltose, maltotriose, maltotetraose, maltohexaose and maltoheptaose. The assays indicated maltotriose as the best acceptor for starch degradation (Figure 7).

### 3.4. Hydrolytic Activity

**Figure 8:**
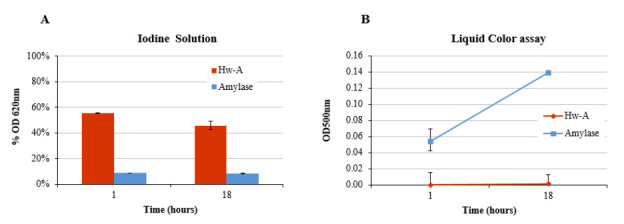
Starch hydrolytic assay. (**A**) Percentages of starch–iodine complex. (**B**) Glucose release measured by the liquid color assay after 1 h and 18 h of incubation.

Hydrolytic assays were carried out to verify the capacity of the enzyme to produce glucose units using different substrates. A first assay was carried out using only starch at pH 7, in the presence of 1 M NaCl at 40 °C, for both 1 h and 18 h of incubation. 10 ng of α-amylase from *B. licheniformis* were used as positive control. In the absence of the acceptor substrate, the enzyme degraded less starch and degraded it more slowly (Figure 8A). In fact, after 1 h of incubation the amount of residual starch (about 55%) was higher than the amounts detectable in the presence of maltooligosaccharides (see Figure 7). Even after 18 h of incubation, the percentage of residual starch was above 40%. The measurement of glucose produced in the reaction, determined by the liquid color assay, indicated that the reaction did not produce glucose monomers, unlike from the reaction with amylase, for which an increase in glucose was observed over time (Figure 8B). Similar hydrolytic assays were carried out using only maltose or maltotriose as substrates. In both cases, Hw-A was not able to hydrolyze the glucosidic bonds, as indicated by the absence of free glucose in the liquid color assays (results not shown).

### 3.5. Thermal Shift Assay

**Figure 9:**
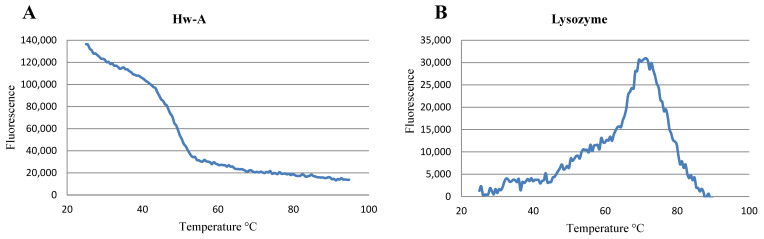
Thermal shift assay. Thermostability curves of Hw-A amylomaltase (**A**) and lysozyme (**B**).

In order to gain information about the correct folding of the recombinant Hw-A protein, a thermal shift assay was carried out using the hydrophobic SYPRO Orange Dye as fluorescent indicator. In an ideal experiment, a properly folded protein has a low number of exposed hydrophobic residues and a relatively low number of fluorescent indicator molecules are bound. By raising the temperature, the increase in the exposed hydrophobic residues that occurs in the course of thermal unfolding enhances dye binding and associated fluorescence. After reaching a maximum fluorescence, further increases in temperature are associated with a fluorescence decrease, due to the aggregation of protein molecules [51]. The inflection of the stability curve (i.e., the fluorescence/temperature curve) indicates the protein melting point, Tm. The thermostability of the amylomaltase was initially investigated using 2.5 μg of protein in 50 mM phosphate buffer pH 7 and 1 M NaCl. A parallel assay was also performed with 2.5 μg of lysozyme as control. The amylomaltase stability curve showed an initial high fluorescence, indicating that the protein was not in ideal conditions for its folding (Figure 9A). After increasing the temperature, the fluorescence values decreased, probably because of the aggregation of protein molecules through their hydrophobic groups. The thermal stability curve obtained for lysozyme showed a Tm at about 72 °C, as reported in the literature [52] (Figure 9B).

**Figure 10:**
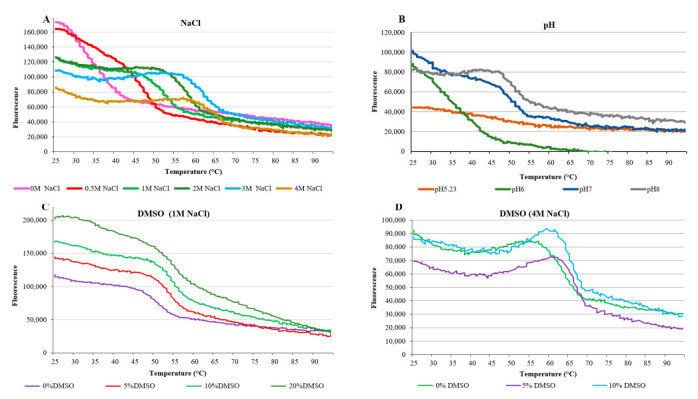
Thermal shift assays for HW-A under different incubation conditions. (**A**) different NaCl concentrations; (**B**) different pH values; (**C**) different concentrations of DMSO in the presence of 1 M NaCl; (**D**) different concentrations of DMSO with 4 M NaCl.

To detect better conditions for the protein folding, additional amylomaltase thermal shift assays were performed by varying some parameters, such as NaCl concentration, pH and DMSO concentration (Figure 10). The assays with DMSO were carried out in the presence of 1 M and 4 M NaCl, respectively.

The assays showed that by increasing the NaCl concentration, the initial fluorescence values decrease and the curves begin to form a fluorescence peak, which shifts towards higher temperatures (Figure 10A). This indicates that high salt concentrations promote correct protein folding and an increase in thermostability. The effect of pH on fluorescence also showed that at high pH values (7 and 8) the curves progressively take on a more regular trend, increasing the thermostability of the protein (Figure 10B). These results were in accordance with the activities of the enzyme observed under different reaction conditions (Figure 4). The data obtained by using different concentrations of NaCl and DMSO showed a more complex relationship with fluorescence, perhaps as the result of contrasting effects of the two substances. In the presence of 1 M NaCl, the increase in DMSO concentration had a positive correlation with initial fluorescence (Figure 10C). However, it is not possible to attribute the increases in fluorescence to further misfolding of the protein or to a reduced ability of water to quench the dye fluorescence, due to its lower abundance in the 20% DMSO solution. At 4 M NaCl concentration, a condition in which the protein showed better folding (Figure 10A), 5% DMSO appeared as an optimal concentration with respect to 0 and 10%, as judged by the relatively low initial fluorescence (Figure 10D).

In any case, the thermal shift assays indicated a possible incorrect folding of the expressed protein. This might be the consequence of the expression of a halophile enzyme by means of a mesophilic bacterium.

## 4. Conclusions

The first identification of an amylomaltase gene from a halophilic organism by functional metagenomics analysis of the saltern of Margherita di Savoia was reported. The gene sequence showed high identity values with amylomaltase genes annotated in the genomes of two strains of the halophilic archaeon *H. walsbyi* strains, isolated in Spain and Australia. Despite the presence of insertion sequences not reported in both archaeal and bacterial homologs, the encoded enzymes have the typical structural domains of functional amylomaltases. The recombinant enzyme was successfully expressed by a conventional *E. coli* expression system in conditions of reduced cell expression activity. It was found active in catalyzing 4-α-glucanotransferase reactions using starch as donor substrate and different maltooligosaccharides as acceptors (with maltotriose giving the greatest activity). The enzyme activity remained nearly the same under conditions of high NaCl concentrations (4 M). High salt concentrations were also effective in producing better folding of the recombinant protein, as indicated by thermal shift assays.

The promising results shown by the enzyme also suggest the possibility of obtaining an enzyme with higher activity by identifying alternative expression systems to *E. coli* and reaction conditions more suitable for an halophilic enzyme. A more active amylomaltase will be useful for studying starch modification reactions under conditions of low water activity, which have not yet been investigated for this group of enzymes.

## Data Availability

Not applicable.

## Supplementary Materials

The following are available online at https://www.mdpi.com/article/10.3390/life12010085/s1, Table S1: Amino acids of conserved regions in amylomaltases of *T. thermophilus* and *H. walsby*; Figure S1: Clustal alignment of Hw-A and GH77 representative sequences; Figure S2: Clustal alignment of Hw-A and 83 archaeal amylomaltases present in the CAZy GH77 family; Figure S3: SDS-PAGE analysis of the fractions obtained from Ni-affinity chromatography.
