# Supplementary material for: Identification of an Amylomaltase from the Halophilic Archaeon Haloquadratum walsbyi by Functional Metagenomics: Structural and Functional Insights
Source: Life (Basel). 2022 Jan 7;12(1):85. doi: 10.3390/life12010085 (PMC8781629; doi:10.3390/life12010085)
Supplement: Supplementary file 1 [file life-12-00085-s001.zip › life-1528815-supplementary.pdf]

Supplementary materials

Identification of an amylomaltase from the halophilic *archaeon Haloquadratum walsbyi* by functional metagenomics: structural and functional insights.

Claudia Leoni[1], Caterina Manzari[2], Tran Hai[3], Peter N. Golyshin[3], Graziano Pesole[1,2], Mariateresa Volpicella[1,2,*] and Luigi R. Ceci[1,*]

[1]Institute of Biomembranes, Bioenergetics and Molecular Biotechnologies, Consiglio Nazionale delle Ricerche, 70126 Bari, Italy;
[2]Department of Biosciences, Biotechnologies and Biopharmaceutics, University of Bari Aldo Moro, 70126 Bari, Italy;
[3]Centre for Environmental Biotechnology, School of Natural Sciences, Bangor University, LL57 2UW Bangor, United Kingdom.
[*] Correspondence: l.ceci@ibiom.cnr.it (L.R.C.); mariateresa.volpicella@uniba.it (M.V.);
Tel.: +39-080-544-3311 (L.R.C. & M.V.)

Content:

Table S1:	**Amino acids of conserved regions in amylomaltases of *T. thermophilus* and *H. walsby*.**
Figure S1:	**Figure S1. Clustal alignment of Hw-A and GH77 representative sequences.**
Figure S2:	**Figure S2. Clustal alignment of Hw-A and 83 archaeal amylomaltases present in the CAZy GH77 family.**
Figure S3:	**Figure S3. SDS-PAGE analysis of the fractions obtained from Ni-affinity chromatography.**

Table S1. Amino acids of conserved regions in amylomaltases of *T. thermophilus* and *H. walsby*.

	T. thermophilus (O87172)	*H. walsbyi* (MZ422727)
ACTIVE SITE		
Catalytic residues	D293<br>E340<br>D395	D345<br>E392<br>D445
i) Four conserved amino acids for substrate binding	Y59<br>D213<br>R291<br>H394	Y61<br>D266<br>R343<br>H444
ii) Other conserved amino acids of the active site	F217<br>W258<br>H294<br>L342<br>N464	Y270<br>W310<br>H346<br>L394<br>N508
iii) Conserved amino acids forming part of the cleft around the active center	S57<br>P58<br>D341<br>G343<br>T393<br>P466	S59<br>P60<br>D393<br>G395<br>T443<br>P510
iv) 250s LOOP	P247<br>P248<br>D249<br>Y250<br>F251<br>S252<br>E253<br>T254<br>G255<br>W302	P300<br>P301<br>T302<br>D303<br>--<br>S304<br>D305<br>D306<br>G307<br>Y354
SUBSTRATE BINDING SITE SECONDARY	Y54<br>Y101<br>Y465	H56<br>R107<br>E509

Figure S1

```
P15977_Escherichia-coli                 ------------------------------------------------------------
Q6R608_Solanum-tuberosum                MVNSGLKSRKVSFRIPYYTQWGQNLLICGSDRLLGSWNVKKGLLLKPSHQGEVLVWSGSI
Q030T7_Lactococcus-lactis               ------------------------------------------------------------
A6YM39_Borrelia-burgdorferi             ------------------------------------------------------------
B5RKZ2_Borrelia-duttonii                ------------------------------------------------------------
A1QYW1_Borrelia-turicatae               ------------------------------------------------------------
O87172_Thermus-thermophilus             ------------------------------------------------------------
UBZ54001_Haloquadratum-walsbyi_MdS      ------------------------------------------------------------

P15977_Escherichia-coli                 ---------------------------MESKRLDNAALAAGISPNYIN--AHGK---P--
Q6R608_Solanum-tuberosum                PVPPGYQSEYSYYVVDDRRNILRWEVGKKRKLLLPDGLQDGQSLELRDLWQTGSDNIPFR
Q030T7_Lactococcus-lactis               ------------------------------------------------------------
A6YM39_Borrelia-burgdorferi             ------------------------------------------------------------
B5RKZ2_Borrelia-duttonii                ------------------------------------------------------------
A1QYW1_Borrelia-turicatae               ------------------------------------------------------------
O87172_Thermus-thermophilus             ------------------------------------------------------------
UBZ54001_Haloquadratum-walsbyi_MdS      ------------------------------------------------------------

P15977_Escherichia-coli                 ---------QSISAETKRRLLDAMHQRTATKVAVTPVPNVMVYTSGKKMPMVVEGSGEY-
Q6R608_Solanum-tuberosum                SAFKDVIFRSSWSLDGER-SLEITQNKLDQD-------GSVILQFRICCPYLEEGTSIYV
Q030T7_Lactococcus-lactis               ------------------------------------------------------------
A6YM39_Borrelia-burgdorferi             ------------------------------------------------------------
B5RKZ2_Borrelia-duttonii                ------------------------------------------------------------
A1QYW1_Borrelia-turicatae               ------------------------------------------------------------
O87172_Thermus-thermophilus             ------------------------------------------------------------
UBZ54001_Haloquadratum-walsbyi_MdS      ------------------------------------------------------------

P15977_Escherichia-coli                 --------SWLLTTEEGTQYKG-------HVTGGKAFNLPTKLPEGY---------HTLT
Q6R608_Solanum-tuberosum                LGSSLNLGQWKIQDGLKLVYAGDSLWQAACVMGKDDFPLKYKYCKHSKAGTSVECGASRE
Q030T7_Lactococcus-lactis               ------------------------------------------------------------
A6YM39_Borrelia-burgdorferi             ------------------------------------------------------------
B5RKZ2_Borrelia-duttonii                ------------------------------------------------------------
A1QYW1_Borrelia-turicatae               ------------------------------------------------------------
O87172_Thermus-thermophilus             ------------------------------------------------------------
UBZ54001_Haloquadratum-walsbyi_MdS      ------------------------------------------------------------

                                                                      β1        β2              α1
P15977_Escherichia-coli                 LTQDDQRAHCRVIVAPKRCYEPQALLNKQKLWGACVQLYTLRSEKNWGIGDFG-DLKAML
Q6R608_Solanum-tuberosum                ISVDVTTGESRFVVLSDGLMREMP----WRGAGVSIPMFSVRSEADLGVGEFL-DLKLLV
Q030T7_Lactococcus-lactis               --------------MVTNTFSIEPYGEKAYHTGIAVPVFSLRTENSSGVGQFS-DLKKLA
A6YM39_Borrelia-burgdorferi             --------------MA-KYKKTRINLNLKRKSGILLNISSLPSKY--GIGDLGKGAYKFI
B5RKZ2_Borrelia-duttonii                ---------------------------MKRKSGILLSISSLPSQY--GIGDLGKGAYEFI
A1QYW1_Borrelia-turicatae               ---------------------------MKRKSGILLNISSLPSKY--GIGDLGRGAYKFI
O87172_Thermus-thermophilus             -------------------------MELPRAFGLLLHPTSLPGPY--GVGVLGREARDFL
UBZ54001_Haloquadratum-walsbyi_MdS      -------------------------MQFDRQAGVFAHITALPGSH--GIGDLGPGARKFL
                                                                        *      ::      *:* :      :
                                                  β3                                    α2
P15977_Escherichia-coli                 VDVAKRGGSFIGLNPIHALYPA-NPESASPYSPSSRRWLNVIYIDVNAVEDFHLSEEAQA
Q6R608_Solanum-tuberosum                DWAVESGFHLVQLLPINDTSVNCMWWDSYPYSSLSVFALHPLYLRVEAISENIPEDIKQE
Q030T7_Lactococcus-lactis               DFTYRSGMDVIQLLPINDTTTFMDWRDSYPYRAISVFALHPLYLDIHEFWKSYTKEQQEK
A6YM39_Borrelia-burgdorferi             DFLFTSSQSYWQMFAYSPID----FTRSPPYSIFSAFAGNVYYIDLEALDKFIDS---D-
B5RKZ2_Borrelia-duttonii                DFLASSSQSYWQILLYSLPN----CSG-VPYLSYSAFAGNISYIDLNAIDRFIDV---D-
A1QYW1_Borrelia-turicatae               DFLADSSQGYWQILPYSPPS----FLE-FPYSSYSAFAGNINYIDLNAIDRFIDI---D-
O87172_Thermus-thermophilus             RFLKEAGGRYWQVLPLGPTG-----YGDSPYQSFSAFAGNPYLIDLRPLAERGYVRLED-
UBZ54001_Haloquadratum-walsbyi_MdS      EWLETANQSVWQFCPLGPTAG---IHDDSPYQSYSAFAGNPLLISLDDLVAEGYLTSED-
                                              .     .                **   *    :   : :  .         :
                                                         β4          α3
P15977_Escherichia-coli                 WWQLPTTQQTLQQARDADWVDYSTVTALKMTALRMAWKGFAQR-----------------
Q6R608_Solanum-tuberosum                IRE------ARVQ-LDKKDVDYEACMATKLSIAKKIFAREKE------------------
Q030T7_Lactococcus-lactis               LLI------LESELNSLEKIDYERCLALKWEYAQIIYQNLAV------------------
A6YM39_Borrelia-burgdorferi             L--------SLLKENETRYSDLKKLS-FKDKFLKEAALNFIN------------------
B5RKZ2_Borrelia-duttonii                L--------GIFGCLEDRCIDYDKLK-TKNIILRDAALNFLH------------------
A1QYW1_Borrelia-turicatae               L--------GTFECLEDRYVDYDKLK-AKEVILRNAALNFLH------------------
O87172_Thermus-thermophilus             ------------PGFPQGRVDYGLLYAWKWPALKEAFRGFKE------------------
UBZ54001_Haloquadratum-walsbyi_MdS      L--------EPVPDFSPHEVVYDTVREYKHDCLQIAAERFRESIGGIAIHPHPHPDPDTT
                                                                    *    :
```

```
                                                                          α4             α5
P15977_Escherichia-coli             --------------------------------DDEQMAAFRQFVAEQGDSLFWQAAFDAL
Q6R608_Solanum-tuberosum            --------------------------------TILNSKSFQEFFSENQEWLKPYAAFCFL
Q030T7_Lactococcus-lactis           --------------------------------KYQKTKSYQQFYKQNEEWLKAYACFSYL
A6YM39_Borrelia-burgdorferi         ------------------------------RASADEVRSFEKFKKKSSYWLLDFASFVAF
B5RKZ2_Borrelia-duttonii            ------------------------------RATIDEMYAFEKFKKSAAYWLLDFSSFVAF
A1QYW1_Borrelia-turicatae           ------------------------------RATIDEIHAFEKFKKSAAYWLLDFSSFVAF
O87172_Thermus-thermophilus         ------------------------------KASPEEREAFAAFREREAWWLEDYALFMAL
UBZ54001_Haloquadratum-walsbyi_MdS  VNSGVDGTDTVPEVTVTLGESVNLSQDTPADSQIEKDAREFKSFYQRENHWLTDYALFMAL
                                                                       .   :  *       *   : *  :
                                                                  α6                α7
P15977_Escherichia-coli             HAQQVKEDEMRWGW---PAWPEMYQNVDSPEVRQFCEEHRDDVDFYLWLQWLAYSQFAAC
Q6R608_Solanum-tuberosum            RNFFETSERSQWGRF--SEFSKEKL----EKLVSKESLHYEVVSFYYYIQFHLHLQLSEA
Q030T7_Lactococcus-lactis           RDINKSANFLAWGKN--ANYDKNLF----DKLKKET----SQLDLYIFVQYLLHSQLTEA
A6YM39_Borrelia-burgdorferi         KEYFFK----ESKNAFNVLFDRGILKKNEKDLFKLRNILSKEIKVQEVLQYFFFSQFQAL
B5RKZ2_Borrelia-duttonii            REYYCRF---QDPQTFNLLFSKEILKRDPKALMRLRETLNVEINIQQVLQYFFFSQFKAL
A1QYW1_Borrelia-turicatae           KEYYSKF---QSSQAFNLLFSREILKRDAKALAKLRETLEVEINIQQVLQYFFFSQFKAL
O87172_Thermus-thermophilus         KGAHGGL---PW-----NRWPLPLRKREEKALREAKSALAEEVAFHAFTQWLFFRQWGAL
UBZ54001_Haloquadratum-walsbyi_MdS  RTSYDG----AW-----TDWPEPIRNRDPDALRDQYESLESDVLYHLIVQFIFDQQWQSL
                                    :                  :           :          :      *:    *
                                                β5            α8                  β6          β7
P15977_Escherichia-coli             WEISQGYEMPIGLYRDLAVGVAEGGAETWCDRELYCLK------ASVGAPPDILGPLGQN
Q6R608_Solanum-tuberosum            AEYAR--KKGVVLKGDLPIGVDRNSVDTWVYPNLFRMN------TSTGAPPDYFDKNGQN
Q030T7_Lactococcus-lactis           VDYCH--KLGIALKGDIAIGIAHDSVDAWTHPELFHLD------KQAGAPPDVFAVNGQN
A6YM39_Borrelia-burgdorferi         KRYAN--DKGIELIMNVPFFIAYDSADVWAYQKYFKLRFDASKDKIAGISPDYFLEQEQA
B5RKZ2_Borrelia-duttonii            KRYAS--NVGIKIISDMPIFMFYDSADVWAHQKYFKLKFDASKDKVTGMPSGNLSYKKYL
A1QYW1_Borrelia-turicatae           KKYAN--NAGIKIVSDIPIFVSYDSADVWAHQKYFKLRFDASKDKVTGVPPDCLFSKKYL
O87172_Thermus-thermophilus         KAEAE--ALGIRIIGDMPIFVAEDSAEVWAHPEWFHLDEEGRPTVVAGVPPDYFSETGQR
UBZ54001_Haloquadratum-walsbyi_MdS  QTVAA--NRGVTLVGDLPIYVALDSADVWATPEIFQLTDTNEPAVVAGVPPTD-SDDGQR
                                       .      : :  :: . :  ...:.*   : : :         .*
                                             α9         α10        β8          β9
P15977_Escherichia-coli             WGLPPMDPHIITARAYEPFIELLRANMQNCGALRIDHVMSMLRLWWIPYGETADQ-----
Q6R608_Solanum-tuberosum            WGFPTYNWEEMSKDNYGWWRARLTQMGKYFTAYRIDHILGFFRIWELPEHAMTGLCGKFR
Q030T7_Lactococcus-lactis           WGFPTYNWEKMAEDGYDWWKKRLTAMSNYFDAYRLDHILGFFRIWQMPENSVRGLLGQFS
A6YM39_Borrelia-burgdorferi         WDSAAYSWNVLKKFKYEWWAKRIGVLRKYADIIKIDHFRGFVSTWEVSAGESYAF-----
B5RKZ2_Borrelia-duttonii            WGNAAYNWKALRKDDYVWWINRIGFMHKYVDIIKFDYFRGFVSTWEISEREFSVYSS---
A1QYW1_Borrelia-turicatae           WGNAAYNWKALRKDDYVWWINRIDFLRKYVDIVRIDYFRGFVSTWEVSAEESLLF-----
O87172_Thermus-thermophilus         WGNPLYRWDVLEREGFSFWIRRLEKALELFHLVRIDHFRGFEAYWEIPASCPTAV-----
UBZ54001_Haloquadratum-walsbyi_MdS  WGNPVYDWAYLRNTGYNWWLDRLDRLFDLVDVTRIDHFKGFDSYYAIPIDADNPA-----
                                    *.        :    :  :   :    .     ::*:. .:   : :
                                                              β10                   α11
P15977_Escherichia-coli             ------------------GAY-------------VHY--PV----------------DDL
Q6R608_Solanum-tuberosum            PSIPISQEELESE-----GLWDFNRLTHPYIGQDLLQEKFGASW-TIIASTFLNEYQKGF
Q030T7_Lactococcus-lactis           PALALSAEEIENNYGIPFRQWGIERFILPFIKDWVIDEVFGRDNRDWIIQTFLDYIGDSN
A6YM39_Borrelia-burgdorferi         -----------------NGLW-------------VKS--PG----------------RDF
B5RKZ2_Borrelia-duttonii            -----------------SGQW-------------VKC--PG----------------KDF
A1QYW1_Borrelia-turicatae           -----------------GGQW-------------VKC--PG----------------KDF
O87172_Thermus-thermophilus         -----------------EGRW-------------VKA--PG----------------EKL
UBZ54001_Haloquadratum-walsbyi_MdS  -----------------AGEW-------------HSV--PG----------------YDF
:
                                                                                 β11
P15977_Escherichia-coli             LSIL-----------------------AL-------------------------------
Q6R608_Solanum-tuberosum            YEFKDECNTEKKIASALKSFLETSMFVESEEKLRRKLFDLLQNVALIKDPEDPRKFYPRF
Q030T7_Lactococcus-lactis           YRFKAEFNNQKAIENT-----------QMENWVREGLYKLQENVIFLKDDENSEKYHPRI
A6YM39_Borrelia-burgdorferi         FNFILNEI------K------------DLK------------------------------
B5RKZ2_Borrelia-duttonii            FRNVLSKI------N------------DLE------------------------------
A1QYW1_Borrelia-turicatae           FKQILNEI------N------------DLE------------------------------
O87172_Thermus-thermophilus         FQKIQEVF------G------------EVP------------------------------
UBZ54001_Haloquadratum-walsbyi_MdS  FETVREHI------G------------SLP------------------------------

P15977_Escherichia-coli             ---------------------------------------------ESKRHRCMVIGEDLG
Q6R608_Solanum-tuberosum            NVEDTTSFMDLDQHSQNVLKRLYYDYYFHRQEGLWRDNAFKTLPVLLNSSDMLACGEDLG
Q030T7_Lactococcus-lactis           GLLSTISFREFGDDYKGRLERLYNDYFYGRNYDFWKEKAYEKLPALKNATKMLACGEDLG
A6YM39_Borrelia-burgdorferi         -----------------------------------------------------IWVEDFQ
B5RKZ2_Borrelia-duttonii            -----------------------------------------------------IWVEDLL
A1QYW1_Borrelia-turicatae           -----------------------------------------------------IWVEDLV
O87172_Thermus-thermophilus         -----------------------------------------------------VLAEDLG
UBZ54001_Haloquadratum-walsbyi_MdS  -----------------------------------------------------FIIEDLG
                                                                                            **:
```

```
                                            α12        β12                α13      β13        α14
P15977_Escherichia-coli                 TVPVEIVGKLRSSGVYSYKVLYFENDHEK-TFRAPKAYPEQ--SMAVAATHDLPTLRGYW
Q6R608_Solanum-tuberosum                LIPSCVHPVMQELGLVGLRIQRMPSEPDV-EFGIPSQYNYM--TVCAPSCHDCSTLRAWW
Q030T7_Lactococcus-lactis               MVPDNVPDVMYHLDILRLIIERMPADERF-VSS-LSEVPYL--SVVTTSSHDTSPLRAWW
A6YM39_Borrelia-burgdorferi             NDLEDVSRLRDFFNFPGMKIMNLAFDFDSSNQNLPHNYIKN--CIVYTGSGDNDTIREFI
B5RKZ2_Borrelia-duttonii                EDRGDAFRLRDYFNFPETRIMQFAFDFDSKNLCLPHNYIKN--CVVYTSTNESNTIRQFI
A1QYW1_Borrelia-turicatae               KDRGDTFRLRDYFGFPGTKMMQCAFDFDSANIYLPHNYIRN--CVVYTGTHESNTIRGFI
O87172_Thermus-thermophilus             VITPEVEALRDRFGLPGMKVLQFAFDDGMENPFLPHNYPAHGRVVVYTGTHDNDTTLGWY
UBZ54001_Haloquadratum-walsbyi_MdS      FIDQALHDLREYFDFPGMRVPHYADWCREGDMYQPMHYPPQ--SVAYSSTHDTDTIVGYY
                                                     ..    :                        :   .  :      :
                                                        α15                                   α16
P15977_Escherichia-coli                 ECGDLTLGKTLGLYPDEVVLRGLYQDRELAKQGLLDALHKYGCLPKRAGHKASLMSMTPT
Q6R608_Solanum-tuberosum                EEDEERRHRFF-----------------------QAVMGSDELPPDQCTPE---------
Q030T7_Lactococcus-lactis               EENHDLTQRYY-----------------------NEVMGWYGEAPNYASVE---------
A6YM39_Borrelia-burgdorferi             NSLDDLHKKYI-----------------------FDYLN-------TNENF---------
B5RKZ2_Borrelia-duttonii                NSVDDEHREYI-----------------------FNYFN-------TNEDF---------
A1QYW1_Borrelia-turicatae               NSVDNEHKKYI-----------------------FDYFN-------TSENA---------
O87172_Thermus-thermophilus             RTATPHEKAFM-----------------------ARYLADWGITF-REEEE---------
UBZ54001_Haloquadratum-walsbyi_MdS      DSLSQSQRDCL-----------------------HYNLG-------VDGSE---------
                                                                             :
                                                       β14   α17                             β15
P15977_Escherichia-coli                 LNRGLQRYIADSNSALLGLQPEDWLDMAE----------PVNIPGTSYQYKNWRRKLSAT
Q6R608_Solanum-tuberosum                IVHFVLRQHVEAPSMWSIFPLQDLLALKEDYTTRPAVEETINDPTNPKHY--WRYRVHVT
Q030T7_Lactococcus-lactis               IIQEIIKRNLNSNAMMVILPIQDWLAMSEHFRKENAKSEQINIPADPYHY--WNYRLHCN
A6YM39_Borrelia-burgdorferi             VVWDMIRSAMGSVSDNVIVPMQDYINLGDKFS--------ANIPKSTLDN--CIFRLLES
B5RKZ2_Borrelia-duttonii                VVWDMIRGAMSSVADNVIVSMEDYLDHVVDH---------KNVSGSMLND----FRILSS
A1QYW1_Borrelia-turicatae               IVWDMIRGAMASVANSVIIPMKDYLDLVADFS--------MNISDAMLNN----FRILSG
O87172_Thermus-thermophilus             VPWALMHLGMKSVARLAVYPVQDVLALGSEAR--------MNYPGRPSGN--WAWRLLPG
UBZ54001_Haloquadratum-walsbyi_MdS      INWSIIDAVWRSDAKIGLTTLQDVLGLDSHAR--------FNEPGTASGN--WEWRCTDS
                                        :   :      : :       :* :                *             :
                                                  α18
P15977_Escherichia-coli                 LESMFADDGVNKLLKDLDRRRRAAAKKK----------------------------
Q6R608_Solanum-tuberosum                MESLLNDKDLTKTIKDLVRGSGRFYPQKDLESGQAKLQLGSEHPSQTQKYGLSNK
Q030T7_Lactococcus-lactis               LQALIENQEWTDFLKNFIKESKRAY-------------------------------
A6YM39_B orrelia-burgdorferi            D----LDATLSQNISFLTRLYGRALE------------------------------
B5RKZ2_Borrelia-duttonii                D----LSTDLSKKISNITKLYGRI--------------------------------
A1QYW1_Borrelia-turicatae               D----LSDDLSKKISDITKLYGRT--------------------------------
O87172_Thermus-thermophilus             E----LSPEHGARLRAMAEATERL--------------------------------
UBZ54001_Haloquadratum-walsbyi_MdS      G----LDEDLARRLAGLTLEHVRD--------------------------------
                                              .      :  :
```

Figure S1. Clustal alignment of Hw-A and GH77 representative sequences. Secondary structural elements highlighted in the *T. thermophilus* sequence were according to the 3D structure described by Przylas et al. [1]. In particular, green regions refer to the $(\beta/\alpha)_8$ barrel (subdomain A); the other coloured regions correspond to subdomains B2 (yellow), B1 (orange) and B3 (cyan). The three catalytic amino acids are reported in red. The non conserved amino acids in the sequences from borreliae discussed in the Introduction are shown in blue. The carbohydrate binding domains of *E. coli* [2] and potato [3] are highlighted in red.

1. Przylas, I.; Tomoo, K.; Terada, Y.; Takaha, T.; Fujii, K.; Saenger, W.; Sträter, N. Crystal Structure of Amylomaltase from Thermus Aquaticus, a Glycosyltransferase Catalysing the Production of Large Cyclic Glucans. *Journal of Molecular Biology* **2000**, *296*, 873–886, doi:10.1006/jmbi.1999.3503.
2. Mareček, F.; Møller, M.S.; Svensson, B.; Janeček, Š. A Putative Novel Starch-Binding Domain Revealed by in Silico Analysis of the N-Terminal Domain in Bacterial Amylomaltases from the Family GH77. *3 Biotech* **2021**, *11*, 229, doi:10.1007/s13205-021-02787-8.
3. Lloyd, J.R.; Blennow, A.; Burhenne, K.; Kossmann, J. Repression of a Novel Isoform of Disproportionating Enzyme (StDPE2) in Potato Leads to Inhibition of Starch Degradation in Leaves But Not Tubers Stored at Low Temperature. *Plant Physiology* **2004**, *134*, 1347–1354, doi:10.1104/pp.103.038026.

Figure S2

```
AUV80673.1      ------------------------------------------------------------
QCC51341.1      ------------------------------------------------------------
QSG15659.1      ------------------------------------------------------------
QSG11910.1      ------------------------------------------------------------
QSG08987.1      ------------------------------------------------------------
QSG05483.1      ------------------------------------------------------------
QGN07616.1      MHSIGPEWREEIDDSTKTQYPAGQDRRADEQGPQRDQKRADRPLARHVDEFGRCVEETVH
QSG11921.1      ------------------------------------------------------------
QSG08998.1      ------------------------------------------------------------
QSG05496.1      ------------------------------------------------------------
CCQ33055.1      ------------------------------------------------------------
QSG02731.1      ------------------------------------------------------------
AUX09035.1      ------------------------------------------------------------
QFU81686.1      ------------------------------------------------------------
AWB27107.1      ------------------------------------------------------------
QLC34969.1      ------------------------------------------------------------
ACV11232.1      ------------------------------------------------------------
QPV62520.1      ------------------------------------------------------------
QLH84085.1      ------------------------------------------------------------
QLH79093.1      ------------------------------------------------------------
QAU11732.1      ------------------------------------------------------------
QAY20111.1      ------------------------------------------------------------
QWC20591.1      ------------------------------------------------------------
QUO46499.1      ------------------------------------------------------------
QKG93724.1      ------------------------------------------------------------
QKY17977.1      ------------------------------------------------------------
ACM57093.1      ------------------------------------------------------------
AZQ14643.1      ------------------------------------------------------------
ATW89261.1      ------------------------------------------------------------
ABQ75827.1      ------------------------------------------------------------
QZX99028.1      ------------------------------------------------------------
QZP38174.1      ------------------------------------------------------------
QZY03180.1      ------------------------------------------------------------
AUG48211.1      ------------------------------------------------------------
AEM58055.1      ------------------------------------------------------------
AHB66799.1      ------------------------------------------------------------
AJF25101.1      ------------------------------------------------------------
AAV46825.1      -----------MDTIYMFAYP---------------------------------RRRVLT
QCP91534.1      ------------------------------------------------------------
QUJ72368.1      ------------------------------------------------------------
UBZ54001.1      ------------------------------------------------------------
CCC40696.1      ------------------------------------------------------------
CAJ52690.1      ------------------------------------------------------------
QCJ48434.1      ------------------------------------------------------------
AHZ21176.1      ------------------------------------------------------------
AFK19479.1      ------------------------------------------------------------
AFK19479.2      ------------------------------------------------------------
QCQ75940.1      ------------------------------------------------------------
AKU07735.1      ------------------------------------------------------------
QOS11852.1      ------------------------------------------------------------
QIB78738.1      ------------------------------------------------------------
ADE05003.1      ------------------------------------------------------------
ADE05003.2      ------------------------------------------------------------
QCC48543.1      ------------------------------------------------------------
ADQ67101.1      ------------------------------------------------------------
QIQ76396.1      ------------------------------------------------------------
QIB74651.1      ------------------------------------------------------------
AIF04697.1      ------------------------------------------------------------
AIF05955.1      ------------------------------------------------------------
QEE14682.1      ------------------------------------------------------------
ADL19304.1      ------------------------------------------------------------
AMD30778.1      ------------------------------------------------------------
AKT33963.1      ------------------------------------------------------------
ABL88479.1      ------------------------------------------------------------
ACB39188.1      ------------------------------------------------------------
AEA11843.1      ------------------------------------------------------------
ABO08194.1      ------------------------------------------------------------
ABP49821.1      ------------------------------------------------------------
AFA40300.1      ------------------------------------------------------------
AAL63326.1      ------------------------------------------------------------
NP_559144.1     ------------------------------------------------------------
AET33957.1      ------------------------------------------------------------
ABD40312.1      ------------------------------------------------------------
QXO95450.1      ------------------------------------------------------------
QVV87979.1      ------------------------------------------------------------
```

```
CCJ37196.1      ------------------------------------------------------------
SAI88308.1      ------------------------------------------------------------
CVK34217.1      ------------------------------------------------------------
BBL68280.1      ------------------------------------------------------------
ABN57193.1      ------------------------------------------------------------
SAI88240.1      ------------------------------------------------------------
CVK34142.1      ------------------------------------------------------------
CCJ37132.1      ------------------------------------------------------------
BBL68006.1      ------------------------------------------------------------
ABN56987.1      ------------------------------------------------------------


AUV80673.1      -------------------------------------------------MTLDRQSGVFC
QCC51341.1      ----------------------------------MCYFQVRAAASREIRMDFDRQSGLFL
QSG15659.1      -------------------------------------------------MDFDRQSGLFL
QSG11910.1      -------------------------------------------------MEFDRQSGLFL
QSG08987.1      -------------------------------------------------MEFDRQSGLFL
QSG05483.1      -------------------------------------------------MEFDRQSGLFL
QGN07616.1      QLREKRGHATTSQPGHLD---AWPGHSRPGVSRRERDSLSRECRQPTGSMAFEKQSGVFL
QSG11921.1      -------------------------------------------------MSFERQSGVFL
QSG08998.1      -------------------------------------------------MSFERQSGVFL
QSG05496.1      -------------------------------------------------MSFERQSGVFL
CCQ33055.1      -------------------------------------------------MSFERQSGVFL
QSG02731.1      ---------------------------------------------------------MFA
AUX09035.1      ------------------------------------------------MTDFDRRSGVFC
QFU81686.1      ------------------------------------------------MAAFDRRSGVFC
AWB27107.1      -------------------------------------------------MALDRESGIFL
QLC34969.1      -------------------------------------------------MNFERTSGVFC
ACV11232.1      -------------------------------------------------MSFDRQSGVFL
QPV62520.1      -------------------------------------------------MQQQRRSGVFL
QLH84085.1      -------------------------------------------------MQQERRSGVFL
QLH79093.1      -------------------------------------------------MQQERRSGVFL
QAU11732.1      -------------------------------------------------MRFDRSDGVFC
QAY20111.1      -------------------------------------------------MRFDRSDGVFC
QWC20591.1      -------------------------------------------------MRFDRSDGVFC
QUO46499.1      -------------------------------------------------MRFDRSDAVFC
QKG93724.1      -------------------------------------------------MRFDRSDAVFC
QKY17977.1      -------------------------------------------------MRFDRSAGVFC
ACM57093.1      -------------------------------------------------MRLDRSSGVFC
AZQ14643.1      -------------------------------------------------MRLDRSGGVFC
ATW89261.1      -------------------------------------------------MDFDRQSGVFL
ABQ75827.1      -------------------------------------------------MQFERQSGVFL
QZX99028.1      -------------------------------------------------MRFDRRSGVLL
QZP38174.1      -------------------------------------------------MRFDRRSGVLL
QZY03180.1      -------------------------------------------------MRFDRRSGVLL
AUG48211.1      -------------------------------------------------MQFERQSGVFL
AEM58055.1      ---------------------------------------------------------MFL
AHB66799.1      -------------------------------------------------MQFERQSGVFL
AJF25101.1      -------------------------------------------------MQFERQSGVFL
AAV46825.1      RIRRRSLLTGRSSPLALPRAAHFVRAPRP-----EKPRPLKCLSGQQVRMQFERQSGVFL
QCP91534.1      -------------------------------------------------MQFERQSGVFL
QUJ72368.1      -------------------------------------------------MQFERQSGVFL
UBZ54001.1      -------------------------------------------------MQFDRQAGVFA
CCC40696.1      -------------------------------------------------MQFDRQAGVFA
CAJ52690.1      -------------------------------------------------MQFDRQAGVFA
QCJ48434.1      -------------------------------------------------MRFDRQSGVFL
AHZ21176.1      -------------------------------------------------MRFSRQSGVFM
AFK19479.1      ----------------------------------------MGLATESVSMRFSRQSGVFM
AFK19479.2      -------------------------------------------------MRFSRQSGVFM
QCQ75940.1      ----------------------------------------MGLATESVSMRFSRQSGVFM
AKU07735.1      -------------------------------------------------MRLSRQSGVFM
QOS11852.1      -------------------------------------------------MRLSRQSGVFM
QIB78738.1      -------------------------------------------------MRLSRQSGVFM
ADE05003.1      -----------------------------------------------MGMRLSRQSGVFM
ADE05003.2      -------------------------------------------------MRLSRQSGVFM
QCC48543.1      -------------------------------------------------MRFDRRSGVFL
ADQ67101.1      -------------------------------------------------MRFDRQSGVFA
QIQ76396.1      -------------------------------------------------MRFDRQSGVFA
QIB74651.1      -------------------------------------------------MRFDRQSGVFA
AIF04697.1      ----------------------------------MAVEGTSKHYGSNGRRPFMRQAGVLC
AIF05955.1      ----------------------------------MAVEGTSKHYGSNGRRPFMRQAGVLC
QEE14682.1      -------------------------------------------------MELPRSAGILL
ADL19304.1      ---------------------------------------------------MERGSGLLL
AMD30778.1      ---------------------------------------------------MERGSGILL
AKT33963.1      ---------------------------------------------------MLRGFGVLL
ABL88479.1      ---------------------------------------------------MLRGFGVLL
ACB39188.1      ---------------------------------------------------MLRGFGVLL
AEA11843.1      ---------------------------------------------------MLRGAGILL
```

```
ABO08194.1        ---------------------------------------------------MLRGAGVLL
ABP49821.1        ---------------------------------------------------MLRGAGVLL
AFA40300.1        ---------------------------------------------------MLRGAGVLL
AAL63326.1        ---------------------------------------------------MLRGAGVLL
NP_559144.1       ---------------------------------------------------MLRGAGVLL
AET33957.1        ---------------------------------------------------MLRGAGILL
ABD40312.1        --------------------------------------------------MKTRGSGILL
QXO95450.1        --------------------------------------------------MKTRGCGTLL
QVV87979.1        --------------------------------------------------MKTRGCGILL
CCJ37196.1        --------------------------------------------------MNRRGSGVLL
SAI88308.1        --------------------------------------------------MNRRGSGVLL
CVK34217.1        --------------------------------------------------MNRRGSGVLL
BBL68280.1        --------------------------------------------------MNRRGSGVLL
ABN57193.1        --------------------------------------------------MNRRSSGVLL
SAI88240.1        -------------------------------------------------MIHTRGSGILL
CVK34142.1        -------------------------------------------------MIHTRGSGILL
CCJ37132.1        -------------------------------------------------MIHTRGSGILL
BBL68006.1        -------------------------------------------------MIQTRGSGILL
ABN56987.1        -------------------------------------------------MIQTRGSGILL
                                                                            : 

AUV80673.1        HVASLPGPQGIGSLGAPARRFVDFLARADQSLWQFCPIGPTTVDYGNSPYSALSAVAGNP
QCC51341.1        HVASLPGPDGIGTLGEPAETFVDFLAESGQSLWQFCPLGPTIPIHDNSPYQSYSAFAGNP
QSG15659.1        HVASLPGPDGIGTLGDPAKTFVDFLADSGQSLWQFCPLGPTIPIHDNSPYQSYSAFAGNP
QSG11910.1        HIASLPGPDGIGTLGDPAETFVDFLADSGQSLWQFCPLGPTIPIHDNSPYQSYSAFAGNP
QSG08987.1        HIASLPGPDGIGTLGDPAETFVDFLADSGQSLWQFCPLGPTIPIHDNSPYQSYSAFAGNP
QSG05483.1        HIASLPGPDGIGTLGGPAETFVDFLADSGQSLWQFCPLGPTIPIHDNSPYQSYSAFAGNP
QGN07616.1        HVSSLPGPDGIGTLGAPAETFVDFLATAEQSLWQTCPIGPTDPDVGNSPYSAYSARAGNP
QSG11921.1        HVSSLSGPDGIGTLGEPAHEFVEYLETAEQSLWQTCPIGPTDPRQGNSPYSSFSARAGNP
QSG08998.1        HVSSLSGPDGIGTLGEPAHEFVEYLETAEQSLWQTCPIGPTDPRQGNSPYSSFSARAGNP
QSG05496.1        HVSSLSGPDGIGTLGEPAHEFVEYLETAEQSLWQTCPIGPTDPRQGNSPYSSFSARAGNP
CCQ33055.1        HVSSLPGPDGIGTLGQPAREFVDFLVESDQSLWQTCPIGPTDAGQGNSPYSAYSARAGNP
QSG02731.1        HPTSLPSSHGIGSIGQPSREFVDTLAEADQSLWQLCPLGPISSIHGNSPYQAYSAFAIEP
AUX09035.1        HPTALPGPGGIGTLGEPARGFLDRITEAGQSLWQLCPLGPTVGIHGNSPYQTCSAFAIDP
QFU81686.1        HPTALPGPHGIGSIGAPARSFVETIAAAGQSLWQLCPLGPTVGIHGDSPYQSSSSFALSP
AWB27107.1        HLSSLPGPHGIGDLGAT-TAVLDWLDRADQSVWQVCPINPTQGVHGHSPYASVSTFAGNP
QLC34969.1        HLTSLPGGHGIGDLGAGARAFLDFLDAADQSLWQFCPLGPTTGVHGHSPYSSPSTFAGNP
ACV11232.1        HLTSLPSPHGIGDLGDGARTFLDFLERAEQSLWQFCPVTPTRGVHGHSPYASPSAFAGNP
QPV62520.1        HLTSLPGPHGVGDLGAGARTFLDFLDRADQSLWQFCPVGPTSDAYGHSPYGSSSAFAGNP
QLH84085.1        HLTSLPGPHGVGDLGAGAREFLDFLDRADQSLWQFCPVGPTSAAYGHSPYGSSSAFAGNP
QLH79093.1        HLTSLPGPHGVGDLGAGAREFLDFLDRADQSLWQFCPVGPTSAAYGHSPYGSSSAFAGNP
QAU11732.1        HVTSLPGAYGIGDLGGGAREFLSFLGDADVDHWQICPIGPTLSVAGESPYQSPSAFAGNP
QAY20111.1        HVTSLPGAYGIGDLGEGARDFLSFLGDADVDHWQICPIGPTLSVAGESPYQSPSAFAGNP
QWC20591.1        HVTSLPGAYGIGDLGQGAREFLSFLGDAGVDHWQICPIGPTLSVAGESPYQSPSAFAGNP
QUO46499.1        HVTSLPGAYGIGDLGEGAREFLSFLGDAGVDHWQICPIGPTISAAGESPYQSPSAFAGNP
QKG93724.1        HVTSLPGAYGIGDLGEGARDFLSFLGDAGVDHWQICPFGPTLSVAGESPYQSPSAFAGNP
QKY17977.1        HVTSLPGRHGIGDLGDGAAAFLSFLGDADVDYWQICPVGPTTDGAGESPYQSPSAFAGNP
ACM57093.1        HVTSLPGRHGIGDLGDGAKAFLSFLGDADIGHWQVCPLGPTMGAAGESPYQSPSAFAGNP
AZQ14643.1        HVTSLPGRHGIGDLGDGAREFLSFLGDADVGHWQICPLGPTIGAAGESPYQSPSAFAGNP
ATW89261.1        HLTSLPGPHGIGDLGDGAREFLSFLQQAEQACWQFCPLGPTSSAHGNSPYQSFSAFAGNP
ABQ75827.1        HITALPGPHGIGDLGEGAHEYIDWLASANQSVWQFCPIGPTASIHGDSPYQSYSAFAGNP
QZX99028.1        HPTALPGPYGIGDLGAGARSFLDFLDRADQSIWQVCPLGPTVGIHGHSPYQTFSGFAGNP
QZP38174.1        HPTALPGPHGIGDLGAGARSFLEFLDRADQSIWQVCPLGPTVGIHGHSPYQTFSGFAGNP
QZY03180.1        HPTALPGPHGIGDLGAGARSFLEFLDRADQSIWQVCPLGPTVGIHGHSPYQTFSGFAGNP
AUG48211.1        HLTSLPGPHGIGDLGSGARAFVDFLDRADQSLWQFCPLGPTASIHGNSPYQSSSAFAGNP
AEM58055.1        HQTSLPGPHGIGDLGAGARAFVDFLDRADQSLWQFCPLGPTASIHGNSPYQSSSAFAGNP
AHB66799.1        HQTSLPGPHGIGDLGAGARAFVDFLDRADQSLWQFCPLGPTASIHGNSPYQSSSAFAGNP
AJF25101.1        HQTSLPGPHGIGDLGAGAWAFVDFLDRADQSLWQFCPLGPTASIHGNSPYQSSSAFAGNP
AAV46825.1        HLTSLPGPHGIGDLGAGARAFVDFLDRADQSLWQFCPLGPTASIHGNSPYQSSSAFAGNP
QCP91534.1        HLTSLPGPHGIGDLGAGARAFVDFLDRADQSLWQFCPLGPTASIHGNSPYQSSSAFAGNP
QUJ72368.1        HLTSLPGPHGIGDLGAGARAFVDFLDRADQSLWQFCPLGPTASIHGNSPYQSSSAFAGNS
UBZ54001.1        HITALPGSHGIGDLGPGARKFLEWLETANQSVWQFCPLGPTAGIHDDSPYQSYSAFAGNP
CCC40696.1        HITALPGPHGIGDLGPGARNFLEWLETANQSVWQFCPLGPTAGIHDDSPYQSYSAFAGNP
CAJ52690.1        HITALPGPHGIGDLGPGARNFLEWLETANQSVWQFCPLGPTAGIHDDSPYQSYSAFAGNP
QCJ48434.1        HVSSLPGPHGIGDLGDGARAFVDFLADADQSLWQFCPLGPTSSAHGNSPYQSFSAFAGNP
AHZ21176.1        HVTSVPGPHGIGDLGDGAHAFVDFLAHAEQSMWQFCPLGPTSPVHANSPYQSASSFAGNP
AFK19479.1        HVTSVPGPHGIGDLGDGAHAFVDFLAHAEQSMWQFCPLGPTSPVHANSPYQSASSFAGNP
AFK19479.2        HVTSVPGPHGIGDLGDGAHAFVDFLAHAEQSMWQFCPLGPTSPVHANSPYQSASSFAGNP
QCQ75940.1        HVTSVPGPHGIGDLGDGAHAFVDFLAHAEQSMWQFCPLGPTSPVHANSPYQSASSFAGNP
AKU07735.1        HVTSVPGPHGIGDLGDGARAFVDFLSDAEQSMWQFCPLGPTEPAMANSPYQSTSSFAGNP
QOS11852.1        HVTSVPGPHGIGDLGDGARAFVDFLSDAEQSMWQFCPLGPTEPAMANSPYQSTSSFAGNP
QIB78738.1        HVTSVPGPHGIGDLGDGARAFVDFLADAEQSMWQFCPLGPTEPAMANSPYQSTSSFAGNP
ADE05003.1        HVTSVPGPHGIGDLGDGARAFVDFLADAEQSMWQFCPLGPTEPAMANSPYQSTSSFAGNP
ADE05003.2        HVTSVPGPHGIGDLGDGARAFVDFLADAEQSMWQFCPLGPTEPAMANSPYQSTSSFAGNP
QCC48543.1        HLTSLPGPHGIGDLGGGARAFVDWLDAAEQSYWQFCPLGPTAPVHGDSPYQAYSAFAGNP
ADQ67101.1        HVPSLPGPHGIGDLGDGARAFIDWLASAEQSLWQFCPLGPTASIHGHSPYQSYSAFAGNP
QIQ76396.1        HVPSLPGPHGIGDLGDGARAFVDWLASAEQSLWQFCPLGPTASIHGHSPYQSYSAFAGNP
QIB74651.1        HVPSLPGPHGIGDLGDGARAFVDWLASAEQSLWQFCPLGPTASIHGHSPYQSYSAFAGNP
```

```
AIF04697.1          HLTSLPSQ----TLGEDAHRFCRLLSQIGCSVWQMLPLTPP--DEHGSPYASYSAFAVWD
AIF05955.1          HLTSLPSQ----TLGEDAHRFCRLLSQIGCSVWQMLPLTPP--DEHGSPYASYSAFAVWD
QEE14682.1          HPTSVYTRYGIGDLGPDTFQFIEFLKASSQHFMQVLPIGPT--GYADSPYQTISAFAGNP
ADL19304.1          HITSLPGSPLVGDLGPSAHEFLEILRDAGQRYWQVLPLSPTAPEHDNSPYSGLSAFAGNP
AMD30778.1          HVTSLPGSPLVGDLGPSAHEFLELLREAGQRYWQVLPLSPTAPEHDNSPYSGLSAFAGSP
AKT33963.1          PVFSLPGGCHVGDMGPEAYKFAEFLADAEATYWQVLPLGHTAPEYDDSPYSALSMLAGNP
ABL88479.1          HISSLPGGCLAGDLGPSAYKFAEFLSKAEATYWQILPLSHTLPEYDDSPYSAVSLLAGNP
ACB39188.1          HISSLPGGCLVGDLGPSAYRFADFLSEAEATYWQILPLSHTLPEYDDSPYSAASLLAGNP
AEA11843.1          PLFSLPGPHGIGDMGPAAYRFVEFLRDAGQTYWQLLPLNPVLPEYDNSPYSSTSSFAGEP
ABO08194.1          HITSLPGGCHVGDLGPEAYAFAEALAYAEQTYWQTLPINHTLPEYENSPYSAASSFAGDP
ABP49821.1          HITSLPGGCYVGDLGPEAYKFAEFLAEAEQTYWQTLPINHSVPEYENSPYSAVSSFAGDP
AFA40300.1          HITSLPGGCYVGDLGPEAYKFAEFLAEAEQTYWQTLPINHSVPEYENSPYSAVSSFAGDP
AAL63326.1          HITSLPGGCFVGDLGPEAYKFADSLAYAEQTYWQTLPINHTVPEYENSPYSAVSSYAGDP
NP_559144.1         HITSLPGGCFVGDLGPEAYKFADSLAYAEQTYWQTLPINHTVPEYENSPYSAVSSYAGDP
AET33957.1          HITSLPGGCYVGDLGPEAYKFAEGLADAGQTYWQTLPINHTLPEYENSPYNAVSSFAGDP
ABD40312.1          HITSLPGQFGIGDLGPQADRFVDFLQASGQRYWQILPVHPTDIRYDNSPYHSISAFAGNP
QXO95450.1          HITSIPTKFGIGDLGPAAYRFVEFLQKAGQRYWQVLPLHPTEHMYDNSPYHALSAFAGNP
QVV87979.1          HITSIPTQFGIGDLGPAAYRFVEFLQKAGQRYWQVLPLHPTENMYDNSPYHALSAFAGNP
CCJ37196.1          HITSLPSVYGIGDLGPAAHRFVDLLAGAGQRYWQILPLNPTCPDLGNSPYLSTSAFAGNT
SAI88308.1          HITSLPSVYGIGDLGPAAHRFVDLLAGAGQRYWQILPLNPTCPDLGNSPYQSTSAFAGNT
CVK34217.1          HITSLPSAYGIGDLGPAAHRFVDLLAGAGQRYWQILPLNPTCPDLGNSPYQSTSAFAGNT
BBL68280.1          HITSLPSVYGIGDLGPAAYRFVDLLAGAGQRYWQILPLNPTCPEFANSPYQGTSAFAGNT
ABN57193.1          HVTSLPSPYGIGDLGPAAYRFVDLLSRAGQRYWQILPLNPTCPEFANSPYQSTSAFAGNT
SAI88240.1          HITSLPSAYGIGDFGPSAYRFVEALAKARQHYWQILPLNPTRVEHASSPYYSPSAFGMNT
CVK34142.1          HITSLPSAYGIGDFGPSAYRFVEALAKARQHYWQILPLNPTRVEHASSPYYSPSAFGMNT
CCJ37132.1          HITSLPSAYGIGDFGPPAYRFVEALAKARQHYWQILPLNPTRIEHASSPYYSPSAFGMNT
BBL68006.1          HITSLPSPYGIGDFGPAAYRFVEALERARQHYWQILPLNPTEAAYAHSPYSSPSAFAANT
ABN56987.1          HITSLPSPYGVGDFGPAAYRFVDALERGRQHYWQILPLNPTETAYAHSPYSSPSAFAANI
                       ::        :*         :        *  *.         ***   *  .

AUV80673.1          LFVDLADLRERGWLERVEGE---------------------PFDDECVEYERVTDHVETH
QCC51341.1          LFVDLDRLVERGWLDELD-------------RP--------DFDDGHVEYGPVREYKEAR
QSG15659.1          LFIDLDELVERGWLEDLD-------------RP--------DFDDGNVEYGPVREYKEAR
QSG11910.1          LFLDLDDLVERGWLEELD-------------RP--------DFNDANVEYEPVREFKEAR
QSG08987.1          LFLDLDDLVERGWLEELD-------------RP--------DFDDANVEYEPVREFKEAR
QSG05483.1          LFLDLDDLVERGWLEELD-------------RP--------DFDDANVEYGPVREFKEAR
QGN07616.1          LFIDLDALVDRGWLADLD-------------RP--------GFDEREVEYDRVDAFKQEA
QSG11921.1          LFIELDALVEEGWLDDPD-------------RP--------DFDDREVEYERVMAFKREA
QSG08998.1          LFIELDALVEEGWLDDPD-------------RP--------DFDDREVEYERVMAFKREA
QSG05496.1          LFIELDALAEEGWLDDPD-------------RP--------DFDDREVEYERVMAFKREA
CCQ33055.1          LFIELDPLVEAGWLDEPD-------------RP--------DFDDREVEFGRVRPFKQGA
QSG02731.1          LLIDLDVLVEQELLTPQEVTPTE------------------PFPNEYVDYDAVREFKYPL
AUX09035.1          LLIDLEDLIERGLLSGNRVETER-------DDA--------RLSDDRVDYDAVRKYKRPL
QFU81686.1          LLIDLEALNERGLLTDDALEPDP------ELDH--------ELTDDRVAYDAVRSFKTSR
AWB27107.1          LFVDLTDLVDRGWLSADALAD----------AP--------R--TDRVDYDRVVPFTEDC
QLC34969.1          LLLDLHALRDQGWLTDAELEA----------PA--------AAEPTRVDYEAVAAFKRER
ACV11232.1          LLVDLTALVERGWLDEETLEN----------PP--------G-DPRTVQYDTVTDFKRER
QPV62520.1          LLVDLTDLVDRGYLDESDLDG----------PE--------GTEPGSVNYEVVAPFVRDR
QLH84085.1          LLVDLVDLADRGYLDDSDLDG----------PE--------GAEPGSVNYEAVAPFKRQR
QLH79093.1          LLVDLVDLADRGYLDDADLDG----------PE--------GAEAGSVNYEVVAPFVRDR
QAU11732.1          LLVDLDGLVDDGWLDEGDLRP----------VP--------EFPVDRVDYDAVREYKLPL
QAY20111.1          LFVDPEGLVDDGWLDEGDLRP----------VP--------DFPTDRVDYEAVREYKLPL
QWC20591.1          LFVDPDGLVADGWLDEGDLEP----------VP--------EFPADRVDYDAVREYKLPL
QUO46499.1          LFVDPDGLVDDGWLDEDDLEP----------VP--------DFPTDRVDYDAVREYKLPL
QKG93724.1          LFVDPDGLVADGWLDEGDLTP----------VP--------DFPTDRVDYDAVREYKLPL
QKY17977.1          LLIDLDGLAADGWLDEADLEP----------AP--------EFPTDRVDYAAVREYKLPL
ACM57093.1          LLIDLDGLVDDGWLDESDLEP----------IP--------DFPEDRVDYDAVREYKQPL
AZQ14643.1          LLIDLDGLVDDGWLDESDLTP----------AP--------DFPSDRVDYEAVRGYKEPL
ATW89261.1          LLISLDRLVDDGWLTDEDLQP----------VP--------DFDEHSVDYEAVGDYKRNQ
ABQ75827.1          LLVSLDTLSEQAYFSLNDVSI----------DA--------PDARHEVEYDAVREFKTAA
QZX99028.1          LLIDLVDLAERGYLIDEEIDVDA---GGDGAGV--------DFSLHEVRYDAVESFKLDR
QZP38174.1          LLIDLVDLAERGYLDDEAAPDAG-------ESV--------DFSPHEVNYDAVESVKLDR
QZY03180.1          LLIDLVDLAERGYLEEDELAAEGDSTGEPDRDA--------DFSRHEVHYHAVESVKLDR
AUG48211.1          LLIDLRDLVDRGYLTADEVEP----------PG--------DFSQQQVNYDRVTEFTERR
AEM58055.1          LLIDLRDLVDRGYLTADEVDP----------PE--------DFSQQHVKYDRVTEFKERR
AHB66799.1          LLIDLRDLVDRGYLTADEVDP----------PE--------DFSQQHVKYDRVTEFKERR
AJF25101.1          LLIDLRDLVDRGYLTADEVDP----------PE--------DFSQQHVKYDRVTEFKERR
AAV46825.1          LLIDLRDLTDRGYLTEADTEP----------PD--------DFSGQQVNYDRVTEFKEAR
QCP91534.1          LLIDLRDLTDRGYLTEADTEP----------PD--------DFSGQQVNYDRVTEFKEAR
QUJ72368.1          LLIDLRDLTDRGYLTEADTEP----------PD--------DFSGQQVNYDRVTEFKEAR
UBZ54001.1          LLISLDDLVAEGYLTSEDLEP----------VP--------DFSPHEVVYDTVREYKHDC
CCC40696.1          LIISLDDLVADGYLTSEDLKP----------VP--------DFSPHEVAYDAVREYKHDR
CAJ52690.1          LIISLDDLVADGYLTNEDLKP----------VP--------DFSPHEVAYDAVREYKHDR
QCJ48434.1          LFVDLRDLLDRGLLTDGDLKP----------VP--------EFDPHETDYQRVSEYKRPL
AHZ21176.1          LLVDLTDLEARGWLTADDLEP----------VP--------DFDDHAVEYERVGNYKRSR
AFK19479.1          LLVDLTDLEARGWLTADDLEP----------VP--------DFDDHAVEYERVGNYKRSR
AFK19479.2          LLVDLTDLEARGWLTADDLEP----------VP--------DFDDHAVEYERVGNYKRSR
QCQ75940.1          LLVDLTDLEARGWLTADDLEP----------VP--------DFDDHAVEYERVGNYKRSR
```

```
AKU07735.1       LLIDLTDLVERGWLTEDDLEP----------VP--------DFDPHAVDYERVGEYKRSR
QOS11852.1       LLIDLTDLVERGWLTDDDLEP----------VP--------DFDPHAVDYERVGEYKRSR
QIB78738.1       LLIDLTDLVARGWLTDADLEP----------VP--------DFDPHAVDYERVGEYKRSR
ADE05003.1       LLIDLTDLVARGWLTDADLEP----------VP--------DFDPLAVDYERVGEYKRSR
ADE05003.2       LLIDLTDLVARGWLTDADLEP----------VP--------DFDPLAVDYERVGEYKRSR
QCC48543.1       LLVSLDRLVEAGYLDAADLEP----------VP--------DFSEHEVDYDRVREYKTDR
ADQ67101.1       LLVDLDQLREDGYLTDDDLEP----------VP--------AFSDHEIEYDRVAEYKREQ
QIQ76396.1       LLVDLDQLREDGYLTDDDLEP----------VP--------AFSEHEIEYDRVAEYKREQ
QIB74651.1       LLVDLDQLREDGYLTDDDLEP----------VP--------AFSEHEIEYDRVAEYKREQ
AIF04697.1       RFRDPAMHY-------------------------------------RLDYE---------
AIF05955.1       RFRDPAMHY-------------------------------------RLDYE---------
QEE14682.1       LLISPDGLIQMELITLEEVDQ----------CLKEIKIHDEPSKNLKVNYQNVIDLKEKI
ADL19304.1       IMVSPQLLANEGLLAADELAS----------LP--------RGEPNRVDYGLAYEHRMRL
AMD30778.1       LMVSPQLLAEEGLLTKEEVAS----------IP--------QGPRGRVDYPLAYQHRRRL
AKT33963.1       PLISLDKATRDGILTRAPPRC---------------------QPTDKADYEAAWQIKRRH
ABL88479.1       ALISLEKMAQDGLLVRSPPKC---------------------PSVERTSFAESWELKRRY
ACB39188.1       ALVSLEKMAQLGLAKRAPPSC---------------------PPAERARFAEAWELKRRY
AEA11843.1       AYISLELLAEQGVLRRSASAE---------------------FGAGRADYEAARAYKVKI
ABO08194.1       RLVSLDLMKRDGLIDQVPQC----------------------PPTPRADYQAAWEVKTKA
ABP49821.1       KLISLDLMKREGLIDQVPDC----------------------PPAERVDYAAAWEVKKKA
AFA40300.1       KLISLDLMKREGLIDQVPDC----------------------PTAERADYAAAWEVKKKA
AAL63326.1       VLISLELMKRDGLIDSVPQC----------------------PPAERADYIKAWEVKRKA
NP_559144.1      VLISLELMKRDGLIDSVPQC----------------------PPAERADYIKAWEVKRKA
AET33957.1       VLISLDLMKKDGLVDQAPQC----------------------PATERADYVKAWEVKRAV
ABD40312.1       LLISPEEMVNDGYLDPSDLQE----------GL--------EGPRDEVDYKAVIAYKERL
QXO95450.1       LLISPEMLVKDGYLDESDLEP----------LP--------DFSSDLVDFPAVIAYKNRL
QVV87979.1       LLISPEMLVKDGYLDESDLEP----------LP--------DFSSDLLDFAAVITYKNRL
CCJ37196.1       WLISPEQMVIDGLLGPEDIGD----------PP--------GFPEDRVDYRAVMDYKNNL
SAI88308.1       WLISPEQMVIDGLLAPEDIRD----------PP--------GFPEDRVDYRAVMDYKNSL
CVK34217.1       WLISPEQMVIDGLLAPEDIRD----------PP--------GFPEDRVDYRAVMDYKNSL
BBL68280.1       WLISPEQMVADGLLAPEDIAD----------PP--------GFPEDRVDYRAVLDYKNGL
ABN57193.1       WLISPEQMVADGFLAPEDIAD----------PP--------DFPADRVDYRAVLDYKNGL
SAI88240.1       LLISPDRLVRDGYLRRADLEP----------VP--------GFPEGRVAYEAATWYKDRL
CVK34142.1       LLISPDRLVRDGYLRRADLEP----------VP--------GFPEGRVAYEAVTWYKDRL
CCJ37132.1       LLISPDRLVRDGYLRRADLEP----------VP--------GFPEGRVAYEAVTWYKDRL
BBL68006.1       LLISPEMLIREGYLRKSELEP----------AP--------GFPDGRAAYEAAVWYHERL
ABN56987.1       LLISPEMLVREGYLRKSELEP----------AP--------GFPEERAAYEAAAWYRERL
                    .                                             :

AUV80673.1       LRRAHDGFEER------------------------------------------------A
QCC51341.1       LTEAFETFKAD------------------------------------------------A
QSG15659.1       LEDAFETFKTD------------------------------------------------A
QSG11910.1       LEEAFETFQEA------------------------------------------------A
QSG08987.1       LEEAFETFQEA------------------------------------------------A
QSG05483.1       LEAAFETFQEQ------------------------------------------------A
QGN07616.1       LRDAFAGFEAD------------------------------------------------A
QSG11921.1       LRDAFEGFSDV------------------------------------------------A
QSG08998.1       LRDAFEGFSDV------------------------------------------------A
QSG05496.1       LRDAFEGFSDV------------------------------------------------A
CCQ33055.1       LRNAFAGFEER------------------------------------------------A
QSG02731.1       LRTAFDRFDDD------------------------------------------------R
AUX09035.1       LREAHRNYRDR------------------------------------------------R
QFU81686.1       LRVAFETFERE------------------------------------------------Q
AWB27107.1       LEAAFEGFRDR------------------------------------------------A
QLC34969.1       LRTAFERFEQS------------------------------------------------A
ACV11232.1       LSAAFDGFEAS------------------------------------------------A
QPV62520.1       LRTAHETFAAK------------------------------------------------A
QLH84085.1       LRAAHDTFTAE------------------------------------------------A
QLH79093.1       LRTAYERFAAA------------------------------------------------A
QAU11732.1       LRTAFDRFEER------------------------------------------------A
QAY20111.1       LRTAFEQFEER------------------------------------------------A
QWC20591.1       LRTAFERFEER------------------------------------------------A
QUO46499.1       LRTAFERFEER------------------------------------------------A
QKG93724.1       LRTAFDRFEER------------------------------------------------A
QKY17977.1       LRTAFERFDEE------------------------------------------------A
ACM57093.1       LRTAFERFDEA------------------------------------------------E
AZQ14643.1       LRTAFERFDEA------------------------------------------------G
ATW89261.1       LRTAHEQFRET------------------------------------------------A
ABQ75827.1       LRTAAESFFDEFG-----------------------------------------------
QZX99028.1       LRVAFERFREV------------------------------------------------A
QZP38174.1       LRTAFDRFREV------------------------------------------------A
QZY03180.1       LRTAFERFREV------------------------------------------------A
AUG48211.1       LRTAYENFDES------------------------------------------------A
AEM58055.1       LRTAYERFDES------------------------------------------------A
AHB66799.1       LRTAYERFDES------------------------------------------------A
AJF25101.1       LRTAYERFDES------------------------------------------------A
AAV46825.1       LRDAYAAFEES------------------------------------------------A
QCP91534.1       LRDAYAAFEES------------------------------------------------A
```

```
QUJ72368.1        LRDAYAAFEES------------------------------------------------A
UBZ54001.1        LQIAAERFRESIGGIAIHPHPHPDPDTTVNSGVDGDTVPEVTVTLGESVNLSQDTPADSQ
CCC40696.1        LRIAAKRFQESVGGINIHA----DTDTTASSGVNDDIAVDVTVTLGDSVDPSQNISTHNQ
CAJ52690.1        LRIAAKRLQESVGGINIHA----DTDTTASSGVNDDIAVDVTVTLGDSVDPSQNISTHNQ
QCJ48434.1        LETAFERFESE------------------------------------------------G
AHZ21176.1        LRTAFDRFEED------------------------------------------------A
AFK19479.1        LRTAFDRFEED------------------------------------------------A
AFK19479.2        LRTAFDRFEED------------------------------------------------A
QCQ75940.1        LRTAFDRFEED------------------------------------------------A
AKU07735.1        LRTAFDRFEAE------------------------------------------------A
QOS11852.1        LRTAFDRFEAE------------------------------------------------A
QIB78738.1        LRAAFDRFEAE------------------------------------------------A
ADE05003.1        LRAAFDRFEAE------------------------------------------------A
ADE05003.2        LRAAFDRFEAE------------------------------------------------A
QCC48543.1        LRTAAERFRSADGDDGSA------------------------------------RASGSA
ADQ67101.1        LRTAHERFQSE------------------------------------------------A
QIQ76396.1        LRTAHERFQSE------------------------------------------------A
QIB74651.1        LRTAHERFQSE------------------------------------------------A
AIF04697.1        ------------------------------------------------------------
AIF05955.1        ------------------------------------------------------------
QEE14682.1        LKIAFKTFLQKQF---------------------------------------------QQ
ADL19304.1        LRLAFSRFKPS-------------------------------------------------
AMD30778.1        LELALSRFKPT-------------------------------------------------
AKT33963.1        LEEAFEKRRGW-------------------------------------------------
ABL88479.1        VEEAFETKLGW-------------------------------------------------
ACB39188.1        LEEAFEGRLGW-------------------------------------------------
AEA11843.1        I----AEAERP-------------------------------------------------
ABO08194.1        LEKAYKRHRRL-------------------------------------------------
ABP49821.1        LEKALRRGKKL-------------------------------------------------
AFA40300.1        LEKALRRGKKL-------------------------------------------------
AAL63326.1        LEKALKRAGRL-------------------------------------------------
NP_559144.1       LEKALKRAGRL-------------------------------------------------
AET33957.1        LEKALKKARAL-------------------------------------------------
ABD40312.1        FDMAYNRFAYF-------------------------------------------------
QXO95450.1        FDMAYNRFVFF-------------------------------------------------
QVV87979.1        FDMAYNRFVFF-------------------------------------------------
CCJ37196.1        FDRAFERFRER-------------------------------------------------
SAI88308.1        FDRAFERFRER-------------------------------------------------
CVK34217.1        FDRAFERFRER-------------------------------------------------
BBL68280.1        YDKAFERFKQQ-------------------------------------------------
ABN57193.1        FDTAFERFKGR-------------------------------------------------
SAI88240.1        FSIAYQRFLHS-------------------------------------------------
CVK34142.1        FSIAYQRFLHS-------------------------------------------------
CCJ37132.1        FSIAYQRFLHS-------------------------------------------------
BBL68006.1        FAAAYERFRYS-------------------------------------------------
ABN56987.1        FAAAYERFRHS-------------------------------------------------


AUV80673.1        TAVERKAFTTFRE-SNDWLDDYALFRALKRDHDSAAWTEWPRGERDRDPDALGAARGRLS
QCC51341.1        SDDEQSNFETFVEESGEWLDEYALYRALRDHYDGVSWLDWPEEAKMRDPDALAEYREELA
QSG15659.1        SDDDRADFEAFTDDAGDWLDEYALYRALRTHFDGVSWLDWPEGAKMRDPEVLAEYREELS
QSG11910.1        SDDERAAFEQFKDDAGEWLEEYALYRALRTHFDGVSWLDWPEKAKMRDPDALEQYREQLA
QSG08987.1        SDDERAAFEQFKDDAGEWLEEYALYRALRTHFDGVSWLDWPEEAKMRDPDALERYREELA
QSG05483.1        GDDERAAFEQFKDDAGEWLDEYALYRALRTHFGGVSWLDWPEEAKTRDPDALEQYREQLA
QGN07616.1        EPAAKTDFETYCGEAGDWLEEYALFRALNDHFDG-EWVEWPAEAKHRDPDALDRYREELA
QSG11921.1        DPDDLTAFESFCEENAEWLDDYALFRALDDHFDADTWLDWPDGAKFRDPDALERYREQLA
QSG08998.1        DPDDLTAFESFCEENAEWLDDYALFRALDDHFDADTWLDWPDGAKFRDPDALERYREQLA
QSG05496.1        DADDWAAFESFCEENAEWLDDYALFRALDDHFDADTWLDWPDGAKFRDPDALERYREQLA
CCQ33055.1        DEDARAAFETFRTENAEWLDDYALFRALDAHFEADTWMDWPDDAKFRDPETLDRYREELA
QSG02731.1        PDELWGAFERFRE-ETDWLHNYALFRALKREFDGESWTNWPEPVALREPDALDEYREELA
AUX09035.1        PTDLVESIEAFES-RAEWLDDYALYRALKRRFDERSWTDWPEEFRLREPDALERAREELA
QFU81686.1        PPDLVDGLEQFRA-ESTWLEEYAHFAALKADFDRQAWLDWPEPIRLRDPDALERSCEQRA
AWB27107.1        TDEERATFEAFREREAGWLADYSLFVTLRERFDGQPWADWPAEFASRDPDAIETIRTERA
QLC34969.1        DAGESDAFAAFRERERDWLADYALYRALKRVHGGESWTEWPRELRERDPDALAAAREEHA
ACV11232.1        EADDRAAFEAFREREATWLRDYTLFTALKAAYDGVPWPEWPADLAGRDPPALEAARETHA
QPV62520.1        SGEERAAFESFRERESAWLEDYALYAALKGAHGDAAWTDWPADLVRREADALSAARETHA
QLH84085.1        SEEERAAFAAFREREAAWLEDYALYAALKDAHGDAAWTDWPADLARRDADALAAARETHA
QLH79093.1        SEDERADFAAFRERESAWLADYALYAALSEAHGGAAWTDWPVDLARRDADALAAARENHA
QAU11732.1        TEEDRAALDAFVDDE-PWLADYALFRAVSDARPEDVWTEWPAPLRTRDPEALSGAREDHA
QAY20111.1        TKEDRAALGAFRERE-PWLADYALFRALSDARPEDVWTEWPAPLRTRDPDALAEAREEHA
QWC20591.1        TDADRAAVDEFRERE-PWLPDYALFRALSDARPEDVWTEWPEPLRTRDPEALASAREEHA
QUO46499.1        SEDDRAAVAEFREHE-PWLADYALFRALSDARPEDVWTDWPAPLRTRDPEALADAREEHA
QKG93724.1        TEADRAALDEFRERE-PWLADYALFRALSDARPEDVWTDWPAPLRTRDPEALAEARDEHA
QKY17977.1        GEEAEADLDAFRERE-PWLADYALFRALSEARDEDTWVEWPEPLRTRDPEALAEARERHA
ACM57093.1        AGKAHSEFDAFRERE-PWLDDYALFRAMSGERDDDTWVEWPDPLRTREPEALDEARERHA
AZQ14643.1        AGDAAPDFDAFCERE-SWLDDYALFRALSAERDEATWVDWPDPLRTREPDALADARERHA
ATW89261.1        TDDDYAALDEFRD-EESWVDDYALFRALKQKHDGVAWVDWPEPIRTRDPDALSEAREELS
ABQ75827.1        ---PTESFGRFCESQSAWLDDYALFMSLHDALE-GAWTEWPEPIRTHESAAVEHYRNELE
```

```
QZX99028.1          SDDDRASFAEFRDREADWLADYALFRALKAEFDETLWTEWPEPARTRDPDALAAYREDLA
QZP38174.1          SDDERETFAAFREREADWLADYALFRALKREFDGTLWTEWPEPVRTRDPDALADYRDELA
QZY03180.1          SDGEREAFAAFREREGDWLADYALFRALKREFDGTLWTEWPEPVRTRDPDALADYRDELA
AUG48211.1          AEADRNAFAEFCEREAGWVDDYALFTALKTEFDDAGWMDWPDDIRTREPAAMERYQEELA
AEM58055.1          SDADQQSFAEFCEREAGWLDDYALFTALKAEFDDAGWMDWPDDIRTREPAAVERYRDELA
AHB66799.1          SDADQQSFAEFCEREAGWLDDYALFTALKAEFDDAGWMDWPDDIRTREPAAVERYRDELA
AJF25101.1          SDADQQSFAEFCEREAGWLDDYALFTALKAEFDDAGWMDWPDDIRTREPAAVERYRDELA
AAV46825.1          SDADQQSFTKFCEREAGWLDDYALFTALKAEFDDAGWMDWPDDIRTREPAAMERYQDDLA
QCP91534.1          SDADQQSFTKFCEREAGWLDDYALFTALKAEFDDAGWMDWPDDIRTREPAAMERYQDDLA
QUJ72368.1          SDADQQSFTEFCEREAGWLDDYALFTALKAEFDDAGWMDWSDDIRTREPAAMERYQDDLA
UBZ54001.1          IEKDAREFKSFYQRENHWLTDYALFMALRTSYD-GAWTDWPEPIRNRDPDALRDQYESLE
CCC40696.1          TSIDATAFKPFYHRESHWLTDYALFMALRTSYD-GAWTDWPESIRHRDPDALSDQYESLD
CAJ52690.1          TSIDATAFKSFYRRESHWLTDYALFMALRTSYD-GAWTDWPESIQHRDPDALSDQYESLD
QCJ48434.1          SDDEREAFEAFRERESSWLDDYTLFISLKEAHDHVAWTDWPEGLKTREPDALEQARDDLE
AHZ21176.1          SEDDRDAFEAFREREAHWLEDYALFASLKDEFE-GAWIDWPTELKTRDPDALARARDEHE
AFK19479.1          SEDDRDAFEAFREREAHWLEDYALFASLKDEFE-GAWIDWPTELKTRDPDALARARDEHE
AFK19479.2          SEDDRDAFEAFREREAHWLEDYALFASLKDEFE-GAWIDWPTELKTRDPDALARARDEHE
QCQ75940.1          SEDDRDAFEAFREREAHWLEDYALFASLKDEFE-GAWIDWPTELKTRDPDALARARDEHE
AKU07735.1          AEDDRAALDAFREREAGWLEDYALFAALKDEFR-RAWIDWPTELKTRDPDALADAREEYA
QOS11852.1          AEDDRAALDAFREREAGWLEDYALFAALKDEFR-RAWIDWPTELKTRDPDALADAREEYA
QIB78738.1          TEDARAALDAFREREAGWLDDYALFAALKDEFR-RAWIDWPTELKTRDPDALADARAEYA
ADE05003.1          TEDDRAALDAFREREAGWLDDYALFAALKDEFR-RAWIDWPTELKTRDPDALADARAEYA
ADE05003.2          TEDDRAALDAFREREAGWLDDYALFAALKDEFR-RAWIDWPTELKTRDPDALADARAEYA
QCC48543.1          TEADREAFEAFRERESGWLDDYALFMALRTRYD-GAWTSWPEPIRARDPDALDAHREELA
ADQ67101.1          TTEDREAFDAFREHESSWLDGYALFMALRTRYD-GAWTTWPQEIRTHDEAAINRHREELA
QIQ76396.1          TTEDREAFDAFCERESSWLDGYALFMALRTRYD-GAWTTWPQEIRTHDEEAINRHREELA
QIB74651.1          TTEDREAFDAFCERESSWLDGYALFMALRTRYD-GAWTTWPQEIRTHDEEAINRHREELA
AIF04697.1          ------SVEKWIEKNLHWLEDYALFAVLKYQLDGLPWTQWPAPLRDRDPEAIKEVIDELY
AIF05955.1          ------SVEKWIEKNLHWLEDYALFAVLKYQLDGLPWTQWPAPLRDRDPEAIKEVIDELY
QEE14682.1          NSKLNDSFVKFCEEQAYWLDDFVLFYSLKVANNLQSWITWPKNFALRHKNALKVWIAGHS
ADL19304.1          --G---DYDRFLDRNSWWLEDYALYVSLREHFKGSDWSSWPEGLRRRDRGEMQAWREKLK
AMD30778.1          --G---DYDRFVDSNSWWLSDYALYASLREHFNGSDWGSWPEGLRRRDRKELEAWGQKLK
AKT33963.1          --G---EFEEFAARNSWWLEPYGVYMALREAY-SAPWTRWPKEL-REGV----DLPEELR
ABL88479.1          --R---DYEEFIAENSWWLESYGRYMALRETF-RVPWIQWPEWARRLNT----PLPPKLA
ACB39188.1          --R---DYEEFAARNSWWLEPYGRYMALREAF-GGPWTAWPAWARRPNA----DLPPRLE
AEA11843.1          --K---DLDEFVG-RNEWLEDYALYSALREYF-GRPWVEWPRELRDRDPAALRQWREKLR
ABO08194.1          --K---DFTAFMEEEWEWLGEYATYMALRERH-G-PHP--PRGNAP-------------R
ABP49821.1          --S---DYKNFVE-STPWLEDYAYYMAMRDLY-G-PWPKWPRRDPP-------------G
AFA40300.1          --S---DYKNFAE-STPWLEDYAYYMAMRDLY-G-PWPKWPRKEPP-------------G
AAL63326.1          --S---DYRHFKD-VTPWLDDYAYYMAMRERF-G-PWPVWPKEEPP-------------P
NP_559144.1         --S---DYRHFKD-VTPWLDDYAYYMAMRERF-G-PWPVWPKEEPP-------------P
AET33957.1          --S---DYRNFVD-KTPWLEDYAYYMAMRERH-G-PWGRWPDEAPP-------------R
ABD40312.1          --GSDPRFIDFCSEQAWWLDDYALFTAIRKYYAPAVWSDWPHPLKERDPQALHEFQEKNN
QXO95450.1          --GTDPAFLTFCEEQAWWLDDYALFIAIKKHYPDILWSDWPSGLRNHNENELHEFKKLYH
QVV87979.1          --GTDPAFLTFCEEQAWWLDDYALFSAIKKHYPDILWSDWPSGLRNHNENELHEFKKLYH
CCJ37196.1          --GPDFRYKEFAAGNVSWLDDYAIFVALRERFEGKVWGDWPPDIRDRHQDALHAYGTELA
SAI88308.1          --GPDFRYKEFAAGNVSWLDDYAIFVALRERFEGKVWGDWPPNIRDRHQDALHAYGTELE
CVK34217.1          --GPDFRYKEFAAGNVSWLDDYAIFVALRERFEGKVWGDWPPNIRDRHQDALHAYGTELE
BBL68280.1          --GCDFRYEDFAAGNTSWLNDYAIFVALKGEFPGKAWSDWPTGIRDRREEALRKRGTELA
ABN57193.1          --DSDFRYEDFAADNTSWLNDYAIFMALRERFSGKVWGDWPAGIRDRHDEALRKYGTELA
SAI88240.1          --GPERGYEEFCDENAWWLEDHALFVALKDHFHRQEWSRWPEEVRARQSEALDDLQRRLA
CVK34142.1          --GPERGYEEFCDRNAWWLEDHALFVALKDHFHGLEWSRWPEEVRARQSEALDDLQRRLA
CCJ37132.1          --GPERGYEEFCDENAWWLEDHALFVALKDHFHGQEWSRWPEEVRARQSEALDDLQRRLA
BBL68006.1          --GPDRRFEAFCDGAGWWLDDHALFVALKDHFRGQAWTRWPDEIRAREPEALKEMRELLD
ABN56987.1          --GPDRRFETFCDGTGWWLDDHALFVALKGHFRGQAWNRWPEDIRARETGALKEMRELLD
                              :      *:  .  :  :

AUV80673.1          GEIAFREFCQFCFDQQWSALRSYATQHDVSLVGDVPIYVAGDSADVWANRDVFQLDEEGQ
QCC51341.1          DSIEYRKFLQWVFDTQWNDLKEYANERGVKFVGDMPIYVDLDSADVWANPEIFKLDAERE
QSG15659.1          ETIEYRKFLQWLFDEQWSELKAYANERGVDLVGDMPIYVDLDSADVWANPEIFKLDEQRE
QSG11910.1          ETIEYRQFLQWLFDQQWSALKEYANERGVKLVGDVPIYVDLDSADVWANPEIFKLDEQRE
QSG08987.1          ETIEYRQFLQWLFDQQWSALKEYANERGVKLVGDVPIYVDLDSADVWANPEIFKLDEQRE
QSG05483.1          ETVEYRQFLQWLFDQQWSALKDYANERGVKLVGDVPIYVDLDSADVWANPEIFKLDEQRE
QGN07616.1          EEIRFRKFLQWVFETQWQALRSYADDRGVSLVGDMPIYVGTNSADVWANPELFKLDEDLE
QSG11921.1          RDVRFRQFLQWVFDRQWAELREHAHEHGIELVGDMAIYVGTNSADVWANPEIFKLDEERE
QSG08998.1          RDVRFRQFLQWVFDRQWAELREHAHEHGIELVGDMAIYVGTNSADVWANPEIFKLDEERE
QSG05496.1          RDVRFRQFLQWVFDRQWAELREHAHEHGVELVGDMAIYVGTNSADVWANPEIFKLDEERE
CCQ33055.1          EDVRFRQFCQWRFERQWADLRAYAHERGIDLVGDMPIYVGANSVDVWANPEIFKLDDDRE
QSG02731.1          DEIRYRAFLQFVAARQWDRLRTHAEQRGVQIVGDLPIYVAQDSADVWANPELFELDEDGT
AUX09035.1          NEISYRVFLQAVAHDQWYRLHEYATDRGIDVVGDVPIYVALDSADVWANRELFELDADGE
QFU81686.1          SDIRYHAFVQYLAAEQWTELRDHAAAHGVEIVGDLPIYVALDSADVWANPDIFELEDHGT
AWB27107.1          DRIAYHAFVQWIFDEQWQRFRAAAADRGIDLIGDLPIYVAWDSADVWAHREAFDLRADGH
QLC34969.1          DEVRYHAFVQWRFDEQWRALREAASERDIRLLGDVPMYAALDSADVWANREAFAVDTAGN
ACV11232.1          EAIRYHAFVQWLFDEQWRALRAAADERGISLVGDLPIYVAWDSADVWANPEAFELDDEGG
QPV62520.1          EAVDYHAFVQWVFDTQWRELRAAAAERGIELVGDLPIYVALDSADVWANQEAFRLDDDGR
QLH84085.1          EAVDYHAFVQWVFDAQWRELRAEAAERGVELVGDLPIYVALDSADVWADQDAFQLDEGGD
QLH79093.1          EAVEYHAFVQWVFDAQWRELRAAAADRGVELVGDLPIYVALDSADVWADQDAFQLDDDGR
QAU11732.1          REVRFREFVQWTFDRQWRDLRAVATEEGVSIVGDVPIYVALDSADVWANPEAFQLDEGNR
```

```
QAY20111.1          AEVRFRAFVQWTFDRQWHELRAVAADEGVSIVGDVPIYVALDSADVWASPEAFRLDEGNR
QWC20591.1          REVRFREFVQWTFDRQWRDLRAVAAEEGVSIVGDVPIYVALDSADVWASPEAFQLDEGNR
QUO46499.1          REVRFREFVQWTFDRQWRDLRAVAAEEGVSIVGDVPIYVALDSADVWASPEAFRLDEGNR
QKG93724.1          REIRFREFVQWTFDRQWRDLRAVAAEEGVSIVGDVPIYVALDSADVWASPEAFRLDEGNR
QKY17977.1          TEVRFREFVQWTFDRQWRDMRAVAAEEGVSIVGDVPIYVALDSADVWANPEAFRLDESNR
ACM57093.1          TEVRFGEFLQWMFDRQWRDLRAVAADEGVSIVGDVPIYVALDSADVWANPEAFRLDEGNR
AZQ14643.1          GEIRFREFVQWVFDRQWRELRSVADDEGVSIVGDVPIYVALDSADVWANPEAFRLDEGNR
ATW89261.1          EEIEYQTLIQYWFDKQWQAFKADAEEKGIKLVGDVPIYVGLDSADVWSSPEAFQLDDQNR
ABQ75827.1          SDIAYRKFVQFVFDRQWRELHQKATDAGVTLVGDLPIYVALDSADVWADPEAFELNDAHQ
QZX99028.1          DEIEFRAFCQWTFDRQWRDLRAAAADRGIEILGDLPIYVALDSADVWAAPAAFDLTDDGR
QZP38174.1          DEVDFRAFCQWTFDRQWRDLRADAADRGVEILGDLPIYVALDSADVWAAPEAFDLDDDGR
QZY03180.1          DAVDFRAFCQWTFDRQWRDLRADAADRGVEILGDLPIYVALDSADVWAAPEAFDLDDDGR
AUG48211.1          DDIRYHKFVQWCFDSQWEALAEYAAERDIGLVGDLPIYVGLDSADVWAYPEVFRLDDDHY
AEM58055.1          DDIRYHKFVQWCFDSQWQALAEYAAERDIGLVGDLPIYVGLDSADVWAYPEVFRLDDDHH
AHB66799.1          DDIRYHKFVQWCFDSQWQALAEYAAERDIGLVGDLPIYVGLDSADVWAYPEVFRLDDDHH
AJF25101.1          DDIRYHKFVQWCFDSQWQALAEYAAERDIGLVGDLPIYVGLDSADVWAYPEVFRLDDDHH
AAV46825.1          DDIRYHKFVQWCFDSQWQALADYAAERDIGLVGDLPIYVGLDSADVWAYPEVFRLDDDHH
QCP91534.1          DDIRYHKFVQWCFDSQWQALADYAAERDIGLVGDLPIYVGLDSADVWAYPEVFRLDDDHH
QUJ72368.1          DDIRYHKFVQWCFDSQWQALADYAAERDIGLVGDLPIYVGLDSADVWAYPEVFRLDDDHH
UBZ54001.1          SDVLYHLIVQFIFDQQWQSLQTVAANRGVTLVGDLPIYVALDSADVWATPEIFQLTDTNE
CCC40696.1          SEVIYHLIIQFIFDQQWQSLQAAATDRDITLVGDLPIYVALDSADVWATPDVFQLTKDNE
CAJ52690.1          SEVIYHLIIQFIFDQQWQSLQAAATDRDITLVGDLPIYVALDSADVWATPDVFQLTKDNE
QCJ48434.1          AERRYHAFCQYLFDEQWRDLKSYASDRGISLVGDLPIYVALDSADVWASPEAFDLDENNE
AHZ21176.1          DEIRYREFVQFCFDEQWRDLAAYADEKSVTLVGDLPIYVALDSADVWAAPEAFDLTESHE
AFK19479.1          DEIRYREFVQFCFDEQWRDLAAYADEKSVTLVGDLPIYVALDSADVWAAPEAFDLTESHE
AFK19479.2          DEIRYREFVQFCFDEQWRDLAAYADEKSVTLVGDLPIYVALDSADVWAAPEAFDLTESHE
QCQ75940.1          DEIRYREFVQFCFDEQWRDLAAYADEKSVTLVGDLPIYVALDSADVWAAPEAFDLTESHE
AKU07735.1          DEIRYREFVQFCFDEQWRALADYADEKGVTLVGDLPIYVALDSADVWAAPEAFDLTESNE
QOS11852.1          DEIRYREFVQFCFDEQWRALADYADEKGVTLVGDLPIYVALDSADVWAAPEAFDLTESNE
QIB78738.1          DEIRYREFVQFCFDEQWRALADYADEKGITLVGDLPIYVALDSADVWAAPEAFDLTESNE
ADE05003.1          DEIRYREFVQFCFDEQWRALADYADEKGITLVGDLPIYVALDSADVWAAPEAFDLTESNE
ADE05003.2          DEIRYREFVQFCFDEQWRALADYADEKGITLVGDLPIYVALDSADVWAAPEAFDLTESNE
QCC48543.1          PEVHYREFVQFVFDRQWRDLREYAHERGVDLVGDLPIYVALDSADVWASPEAFDLTPDRE
ADQ67101.1          DEISYREFAQFVFDTQWRELKAYANDHGVKLVGDLPIYVALDSADVWAAPEAFDLTEENE
QIQ76396.1          DEVSYRKFAQFVFDTQWRELKAYANDHGVKLVGDLPIYVALDSADVWAAPEAFDLTEENE
QIB74651.1          DEVSYRKFAQFVFDTQWRELKAYANDHGVKLVGDLPIYVALDSADVWAAPEAFDLTEENE
AIF04697.1          GSMGKWMEEQFIIKIDWSQLRNTAEECGVSLFGDLPFFVAHDSADVWANQHLFKLEASGA
AIF05955.1          GSMGKWMEEQFIIKIDWSQLRNTAEECGVSLFGDLPFFVAHDSADVWANQHLFKLEASGA
QEE14682.1          RELKLYKFIQWIFSIQWTNLRDFAHKNNVEIIGDMPIFVAHDSVDVWANSDLFTLNPDGT
ADL19304.1          ERIELEHFIQYEFFSQWSSLRAHARRLGIRIIGDIPIYVSYDSADVWSNPRIFKLGRDLR
AMD30778.1          SRVELELFTQYEFFSQWSRLRAHARRLGIRIIGDIPIYVSYDSADAWANPRIFKLNRDLR
AKT33963.1          RRAQFYQYVQYVFWSQWLELRRHVNNLGIFIIGDLPIYPAHDSADVWDGRRYFKLAPDGA
ABL88479.1          KLAEFYKYVQYVFWRQWDDLKRYVNSLGIFIIGDLPIYPALDSADVWEGRRYFKLAPDGT
ACB39188.1          RRADFYRYVQFHFWLQWEELKRYVNSLGVFIIGDLPIYPALDSADVWEGQRYFKLAPDGA
AEA11843.1          RRIELEYLLQYFFWTQWRELKRHANSLGIFLIGDLPIYLSLDSADVWAHRELFKLDAEGR
ABO08194.1          DRVEFYAFAQFVFWTQWRRLKEYVNSLGIFLIGDLPYYPGPDSADVWAHPRLFKVGQDGR
ABP49821.1          ELVELYKFAQFVFWRQWRELKQYVNSLGIFLIGDLPIYPSLDSADVWRHRRYFKITEDGA
AFA40300.1          ELVELYKFAQFVFWRQWRELKQYVNSLGIFLIGDLPIYPSLDSADVWRHRRYFKITEDGA
AAL63326.1          ASLELYKFAQFVFWTQWERFKEYVNSLGIFLIGDLPFYPSLDSVDVWRNKKYFKVGEDGR
NP_559144.1         ASLELYKFAQFVFWTQWERFKEYVNSLGIFLIGDLPFYPSLDSVDVWRNKKYFKVGEDGR
AET33957.1          RLVELYKFAQFVFWSQWERLKQYVNDLGILLIGDLPFYPSVDSVDVWRYRQYFKVGDDGK
ABD40312.1          SVIEREKFIQYIFSGQWEALRKKCHNAGIRLIGDIPIYIDYDSSDVWTYSDLFRLDKERR
QXO95450.1          SVIEREQFLQFIFFHQWAALRKRSHELGIWLIGDIPIYVDYDSSDAWTHQHLFQLNEDKK
QVV87979.1          SVIEREQFLQFIFFHQWAALRKRSHELGIWLIGDIPIYVDYDSSDAWTHQHLFQLNEDKK
CCJ37196.1          DRILREKFLQYVFDRQWKTLKNHCRSRHLQVIGDLPVYLTYDSVDLWANPDLFKLDEQKK
SAI88308.1          DRILREKFLQYVFDRQWKTLKNHCRSRHLQVIGDLPVYLTYDSVDIWANPDLFKLDEQKK
CVK34217.1          DRILREKFLQYVFDRQWKTLKNHCRSRHLQVIGDLPVYLTYDSVDIWANPDLFKLDEQKK
BBL68280.1          DRILREKFLQYVFDRQWQALKVHCRARHLQIIGDVPIYVTYDSVDLWANPGLFKLDEQKK
ABN57193.1          DRIFREKFLQYVFDRQWETLKKHCRMRHLQIVGDIPFYLTYDSVDLWANPRLFKLDEQKK
SAI88240.1          DGVRKEKFLQYLAARQWSALHRHCTDLGIQVIGDIPIYVAYDSVDVWQNPGIFKLDEDLR
CVK34142.1          DVVRKEKFLQYLAARQWSALHRHCTDLGVQVIGDIPIYVAYDSVDVWQNPGIFKLDEDLR
CCJ37132.1          DEVRKEKFLQYLAARQWSALHRYCTDRGIQIIGDIPIYVAYDSVDVWQNPGIFKLGEDLR
BBL68006.1          DQIRKEKFLQYIAARQWSALRRYCTDHGIQVIGDIPIYVAYDSVDVWANPGIFKLDEDLR
ABN56987.1          DRIRKEKFLQYIAARQWSALRRYCTERGIQVIGDIPIYVAYDSVDVWAHPGIFKLDEDLR
                             *     :*  :        : ..**:  :   :* * *     * :

AUV80673.1          PTAVAGVPDAPDEVFSAQRWGMPLFDWEALERTGFAWWRSRLERLFAQCDIVRIDHFRGL
QCC51341.1          PKYVSGVPPDE-FSDDGQMWGTPVYDWDALDERDYGWWVKRFERLLQRVDIFRIDHFKGF
QSG15659.1          PKYVSGVPPDE-FSDDGQMWGTPVYDWAALAERDYGWWVSRFERLLQRVDIFRIDHFKGF
QSG11910.1          PEYVSGVPPDE-FSDDGQMWGTPVYDWAALAERDYGWWVSRFERLLQRVDIFRIDHFKAF
QSG08987.1          PEYVSGVPPDE-FSDDGQMWGTPVYDWAALAERDYGWWVSRFERLLQRVDIFRIDHFKAF
QSG05483.1          PEYVSGVPPDE-FSDDGQMWGTPVYDWTALAERDYGWWVSRFERLLQRVDIFRIDHFKAF
QGN07616.1          PAYVAGVPPDE-FSETGQLWGMPVYDWAAHAEDEYEWWLQRFEGLLDRFDVFRIDHFKGF
QSG11921.1          PIYVSGVPPDD-FSDTGQLWGTPVYDWETLADREYDWWIQRFAGLLDRFDVFRIDHFKGF
QSG08998.1          PIYVSGVPPDD-FSDTGQLWGTPVYDWETLADREYDWWIQRFAGLLDRFDVFRIDHFKGF
QSG05496.1          PIYVSGVPPDD-FSDTGQLWGTPVYDWETLADREYDWWIQRFAGLLDRFDVFRIDHFKGF
CCQ33055.1          PIYVAGVPPDD-FSDTGQLWGMPVYDWETLAERDYDWWVQRFAGLLDRFDVFRIDHFKGF
QSG02731.1          PKDVSGVPADE--VNPAQKWGPPVYDWAEHEASGYEWWIARLERQLELADIVRLDHFRGF
```

```
AUX09035.1        PAVVAGVPPGD--DDDGQKWGNPVYDWDALAATGYEWWVDRMAWTLDLVDLVRLDHFRGF
QFU81686.1        PAVVSGVPPGGGETDGQKWGNPVYDWDRLAETSYEWWVDRFERLFELVDIARLDHFLAF
AWB27107.1        PAAVAGVPPNP--GDDGQRWGMPVYDWDALA-PEYDWWVARLDRLLALVDRVRIDHFKAF
QLC34969.1        PEAVAGVPPSA--GDDGQRWGMPVYDWDALAADDYGWWVRRLDRLFDLVDVARLDHFKAF
ACV11232.1        PTAVAGVPPNP--GDDGQRWGNPVYDWETLRAEDYGWWRDRLDRLLSLVDIARIDHFKAF
QPV62520.1        PTAVAGVPPNP--GDDGQRWGNPVYDWGRLRETDYEWWRDRLSRLLDLVDLARIDHFKAF
QLH84085.1        PTAVAGVPPNP--GDDGQRWGNPVYDWDRLRETDYEWWRRRLSRLLDLVDLARIDHFKAF
QLH79093.1        PTAVAGVPPNP--GDDGQRWGNPVYDWDRLRETDYEWWRRRLSRLLDLVDLARIDHFKAF
QAU11732.1        PTVVAGVPPNP--GDDGQRWGNPVYDWERLSESGYDWWLDRFRRLFELADVARLDHFLGF
QAY20111.1        PTAVAGVPPNA--GDSGQRWGNPLYDWERLAESGYDWWLDRFRRLFDLADVARLDHFLGF
QWC20591.1        PTAVAGVPPNA--GDSGQRWGNPLYDWERLAESGYDWWLDRFRRLFELADVARLDHFLGF
QUO46499.1        PTAVAGVPPNA--GDSGQRWGNPLYDWEHLADTGYDWWLDRFRRLFELADVARLDHFLGF
QKG93724.1        PTAVAGVPPNA--GDDGQRWGNPLYDWEHLAESGYDWWLDRFRRLFELADVARLDHFLGF
QKY17977.1        PTAVAGVPPNP--GDDGQRWGNPVYDWERLAERDYDWWIARFRRLFDLADVARLDHFLGF
ACM57093.1        PAVVAGVPPNP--GDDGQRWGNPVYDWDRLAERDYDWWIARFRRLFELADVARLDHFLGF
AZQ14643.1        PTAVAGVPPNP--GDDGQRWGNPVYDWDRLAERDYDWWIDRFRRLFDLADVARLDHFLGF
ATW89261.1        PTAVAGVPPNA--GDSGQKWGNPLYDWETLADNDYDWWGRRLGRLFEQVDITRLDHFQGF
ABQ75827.1        PSAVAGVPPTE--SDAGQRWGNPLYDWEHLRETNYEWWISRLERLFELVDITRIDHFKGF
QZX99028.1        PAAVAGVPPNA--GDSGQRWGNPVYDWDHLAETGYEWWIDRLRRLFDTVDYARLDHFKGF
QZP38174.1        PAAVAGVPPNM--GDSGQRWGNPVYDWDRLAETGYEWWLDRLRRLFDLVDYARLDHFKGF
QZY03180.1        PAAVAGVPPNM--GDSGQRWGNPVYDWNRLAETGYEWWLDRLRRLFDLVDYARLDHFKGF
AUG48211.1        PAEVAGVPPNP--GDDGQRWGNPVYDWETLRANDYGWWVDRFERLFDLVDIARIDHFKGF
AEM58055.1        PAEVAGVPPNP--GDDGQRWGNPVYDWDALRANDYGWWVDRFERLFDLVDIARIDHFKGF
AHB66799.1        PAEVAGVPPNP--GDDGQRWGNPVYDWDALRANDYGWWVDRFERLFDLVDIARIDHFKGF
AJF25101.1        PAEVAGVPPNP--GDDGQRWGNPVYDWDALRANDYGWWVDRFERLFDLVDIARIDHFKGF
AAV46825.1        PAEVAGVPPNP--GDDGQRWGNPVYDWDALRANDYGWWVDRFERLFDLVDIARIDHFKGF
QCP91534.1        PAEVAGVPPNP--GDDGQRWGNPVYDWDALRANDYGWWVDRFERLFDLVDIARIDHFKGF
QUJ72368.1        PAEVAGVPPNP--GDDGQRWGNPVYDWDALRANDYGWWVDRFERLFDLVDIARIDHFKGF
UBZ54001.1        PAVVAGVPPTD--SDDGQRWGNPVYDWAYLRNTGYNWWLDRLDRLFDLVDVTRIDHFKGF
CCC40696.1        PAVVAGVPPSD--GDDGQRWGNPVYDWEHLRETEYNWWLHRLDRLFTLVDITRIDHFKGF
CAJ52690.1        PAVVAGVPPSD--GDDGQRWGNPVYDWEHLRETEYNWWLHRLDRLFTLVDITRIDHFKGF
QCJ48434.1        PAAVAGVPPQF--GDSGQRWGNPLYDWDHLRETDYEWWRRRFSRLFDLVDVARLDHFKAF
AHZ21176.1        PAVVAGVPPNP--NDSGQRWGNPLYDWDTLQEDDYSWWLDRLRRLFDLVDVTRIDHFKGF
AFK19479.1        PAVVAGVPPNP--NDSGQRWGNPLYDWDTLQEDDYSWWLDRLRRLFDLVDVTRIDHFKGF
AFK19479.2        PAVVAGVPPNP--NDSGQRWGNPLYDWDTLQEDDYSWWLDRLRRLFDLVDVTRIDHFKGF
QCQ75940.1        PAVVAGVPPNP--NDSGQRWGNPLYDWDTLQEDDYSWWLDRLRRLFDLVDVTRIDHFKGF
AKU07735.1        PAVVAGVPPNP--NDSGQRWGNPLYDWETLREDGYGWWLDRLRRLFDLVDVTRIDHFKGF
QOS11852.1        PAVVAGVPPNP--NDSGQRWGNPLYDWETLREDGYGWWLDRLRRLFDLVDVTRIDHFKGF
QIB78738.1        PAVVAGVPPNP--DDAGQRWGNPLYDWETLREDGYGWWLDRLRRLFDLVDVTRIDHFKGF
ADE05003.1        PAVVAGVPPNP--DDAGQRWGNPLYDWETLREDGYGWWLDRLRRLFDLVDVTRIDHFKGF
ADE05003.2        PAVVAGVPPNP--DDAGQRWGNPLYDWETLREDGYGWWLDRLRRLFDLVDVTRIDHFKGF
QCC48543.1        PAAVAGVPPNP--QDDGQRWGNPVYDWETLREADYDWWLARLDRLFDLVDVTRIDHFKGF
ADQ67101.1        PVAVAGVPPNP--NDDGQRWGNPLYDWDYLRENDYDWWMDRLERLFELVDVTRIDHFKGF
QIQ76396.1        PVAVAGVPPNP--NDDGQRWGNPLYDWDYLRENDYDWWMDRLARLFELVDVTRIDHFKGF
QIB74651.1        PVAVAGVPPNP--NDDGQRWGNPLYDWDYLRENDYDWWMDRLARLFELVDVTRIDHFKGF
AIF04697.1        PSVVAGVPPDY-FSEDGQKWGTVLYNWDAHRDEDFAWWKARTARMFEMFDLVRIDHFRAL
AIF05955.1        PSVVAGVPPDY-FSEDGQKWGTVLYNWDAHRDEDFAWWKARTARMFEMFDLVRIDHFRAL
QEE14682.1        LEYQAGVPPDY-FSKTGQLWGNPLYKWEILQERNFKWFIQRFKRQIELFDWIRVDHFRGF
ADL19304.1        PTYVSGVPPDY-FSRTGQLWNTPVYDWESLRRERYSWWIRRFAHASELFDAVRLDHFRGF
AMD30778.1        PAFVSGVPPDY-FSRTGQLWNTPVYDWEELRRERYSWWVKRMAHASRLFDAVRLDHFRGF
AKT33963.1        PLYVSGVPPDY-FSPTGQLWGTPVYDWDAVRRDRYAWWARRLRRMLEAFDYIRLDHFRGY
ABL88479.1        PLYVSGVPPDY-YSPTGQLWGTPVYNWEELRRDRYVWWTRRLTRMLSLFDYLRLDHFRGY
ACB39188.1        PLYVSGVPPDY-YSPTGQLWGTPVYNWAELRRDRYVWWTRRLTRLLSIFDYIRLDHFRGY
AEA11843.1        PLYVAGVPPDY-FSPTGQLWGNPVYDWDAMRKEGFRWWLDRLRYTLSVFDYVRLDHFRGY
ABO08194.1        PLYVGGVPPDY-FSPTGQLWGNPVYNWEEMEREGYRWWIRRLKHALRTFDYVRLDHFRGY
ABP49821.1        PLYVAGVPPDY-FSPTGQLWGNPVYNWEALRADGYRWWLDRLRHTLSAFDYVRLDHFRGY
AFA40300.1        PLYVAGVPPDY-FSPTGQLWGNPVYNWDALRADGYRWWLDRLRHALSVFDYVRLDHFRGY
AAL63326.1        PLYVAGVPPDY-FSATGQLWGNPVYNWEALERDGFKWWVNRLKHAVTVFDYVRLDHFRGY
NP_559144.1       PLYVAGVPPDY-FSATGQLWGNPVYNWEALERDGFKWWVNRLKHAVTVFDYVRLDHFRGY
AET33957.1        PLYVAGVPPDY-YSATGQLWGNPVYNWEALERDGYRWWVERLRHMLEVFDYVRLDHFRGY
ABD40312.1        PTFVAGVPPDY-FSATGQVWNNPLYDWDAIKNSGFSWWLLRLKRELSMVDYVRIDHFRGL
QXO95450.1        PLYVAGVPPDY-FSATGQVWNNPIYDWDEMKKSGFSWWVKRMEHELKFVDYVRIDHFRGL
QVV87979.1        PLYVAGVPPDY-FSATGQVWNNPIYDWDEMKKTGFSWWVKRMEHELKFVDYVRIDHFRGL
CCJ37196.1        PAVVAGVPPDA-FSATGQLWGNPVYNWEAHLEQEFAWWESRIDRTLALVDRVRLDHFRGF
SAI88308.1        PIVVAGVPPDA-FSATGQLWGNPVYNWEAHQEQGFAWWESRIDRTLALVDRVRLDHFRGF
CVK34217.1        PIVVAGVPPDA-FSATGQLWGNPVYNWEAHQEQGFAWWESRIDRTLALVDRVRLDHFRGF
BBL68280.1        PTAVSGVPPDA-FSETGQLWGNPVYNWDAHREQGFAWWESRIDRTLALVDRLRLDHFRGF
ABN57193.1        PTAVAGVPPDM-FSDAGQLWGNPVYNWDAHRERGFGWWESRIDRTLALVDRLRLDHFRGF
SAI88240.1        PTVVAGVPPDM-FSKTGQLWGNPVYDWPALRDRGYDWWIRRIRRSAELYDLFRIDHFRAF
CVK34142.1        PTVVAGVPPDM-FSKTGQLWGNPVYDWPALRDRGYDWWIRRIRRSAELYDLFRIDHFRAF
CCJ37132.1        PTVVAGVPPDM-FSETGQLWGNPVYDWPALRDRGYDWWMRRIRRSAELYDLFRIDHFRAF
BBL68006.1        PTVVAGVPPDI-FSKTGQLWGNPVYDWAALRKRGYNWWAGRLARSGELYDLFRIDHFRAF
ABN56987.1        PTVVAGVPPDI-FSRTGQLWGNPVYDWAALRERGYDWWAGRIARSAELYDLFRIDHFRAF
                      .***        .* *.  ::.*       : *:  *        *  *:*** .

AUV80673.1        ERYWAIPADESDPNVGEWRDVPSEAFFDALSRE----FGDLSARAFAEDIGHPTDAVDDL
QCC51341.1        ESYWEIPADAETAREGEWVEGPHEDVFYAIRDE----LGDLP--IVVEDLGEITPKMEEI
QSG15659.1        ESYWEIPADADTAREGEWVDGPHEDIFYAVRDE----LGELP--IVVEDLGEITDEMDRI
```

```
QSG11910.1        ESYWEIPADADTAREGEWVVGPHEEVFYAVRDQ----LGDLP--IVVEDLGEITEEMDRI
QSG08987.1        ESYWEIPADADTAREGEWVVGPHEEVFYAVRDQ----LGDLP--IVVEDLGEITEEMDRI
QSG05483.1        ESYWEIPADADTAREGEWVDGPHEEVFYAVRDQ----LGDLP--IVVEDLGEITEEMDRI
QGN07616.1        ESFYQIPADHDTAVDGEWVTGPGEALFQTVEAE----LGELP--IIVEDLGHITEETRAL
QSG11921.1        ESYYQVPAEEDTAMNGEWVSGPGRDFFEAVRER----LGDLP--IVVEDLGEVTDATRAL
QSG08998.1        ESYYQVPAEEDTAMNGEWVSGPGRDFFEAVRER----LGDLP--IVVEDLGEVTDATRAL
QSG05496.1        ESYYQVPAEEDTAMNGEWVSGPGRDFFEAVRER----LGDLP--IVVEDLGEVTDATRAL
CCQ33055.1        ESYYQVPAEEDTAMNGEWVSGPGRDFFAAVRDQ----LGELP--IVVEDLGAITDATREL
QSG02731.1        EEYYAIPADGDG-DEGEWRDGPGIDFFDRIEDE----LGELP--FIAEDIGFITEEITQL
AUX09035.1        ESFWAVPVDAPA-REGEWRPGPGRDLFETIERAVETVSEGLP--AIAENLGHVTDEVETL
QFU81686.1        DRYWAIPADAPA-HEGEWRDGPGRELFETVADG----CDELP--LIAEDLGHLTDGVERL
AWB27107.1        ESFWAIPAEKSDPAAGEWRAGPGAAFFERVRAE----HGALP--FIVEDLGFLDDALATL
QLC34969.1        DAYWAIPANAASAADGEWHPGPGAAFFERVRED----LGDLP--FVVEDLGFLDDGVLGL
ACV11232.1        DEYWAIPADADDPAAGEWRPGPGADFFETIRAE----LGELP--FVVEDLGFLDESMVAL
QPV62520.1        DEYWAIPADADDPAAGEWRPGPADDFFETVREH----LGGLP--FVVEDLGFLDESVVGL
QLH84085.1        DEYWAIPADADDPAAGEWRPGPADDFFETVDEH----LGGLP--FVVEDLGFLDESMVAL
QLH79093.1        DEYWAIPADADDPAAGEWRPGPADGFFEAMRAD----LGGLP--FVVEDLGFLDESVVAL
QAU11732.1        VTYWAIPEDSDDPADGEWREGPGRDLFETVERE----LGEAP--FIAEDLGFEEPAMDEL
QAY20111.1        VKYWAIPADSDDPADGEWRDGPGRDLFEAVERE----LGAAP--FIAEDLGFEDPAMDEL
QWC20591.1        VKYWAIPADGGDPADGEWREGPGRDLFEAVERE----LGEAP--FIAEDLGFEEPAMDEL
QUO46499.1        VNYWAIPADSDDPADGEWRDGPGRDLFETVERE----LGEAP--FIAEDLGFEEPAMDEL
QKG93724.1        AKYWAIPADSDDPADGEWRDGPGRDLFETVERE----LGEAP--FIAEDLGFEEPAMEEL
QKY17977.1        VKYWAIPADRDDPAAGEWRAGPGRDLFETVERE----LGQTP--FIAEDLGFEEPAMDEL
ACM57093.1        VKYWAIPADADDPAAGEWCEGPGRDLFETVERE----LGQAP--FIAEDLGFEEPAMDEL
AZQ14643.1        VKYWAIPADADDPAAGEWCDGPGRALFETVERE----LGQAP--FIAEDLGFEEPAMNEL
ATW89261.1        LEYWAIPTEADSAAEGEWRDGPGAAFFEAIEAQ----LGELP--FIAEDLGFPDAELQAL
ABQ75827.1        ESFWSIPADADSPGAGEWKSGPGKDFFDTVAAR----LGQLP--FIVEDLGHIDQSLHEL
QZX99028.1        DEFWAIPADSDDPADGEWREGPGDAFFETVREE----FDRLP--FVAEDLGFLDAGSAAL
QZP38174.1        DEFWAIPADSDDPADGEWREGPGEAFFEAVRGE----FGRLP--FVAEDLGFLDAESAGL
QZY03180.1        DEFWAIPADSDDPADGEWREGPGEAFFEAVRGE----FGRLP--FVAEDLGFLDAESAGL
AUG48211.1        DEFWSIPAEADDPAAGQWRDGPGAHFFETVRDR----LGELP--FFVEDLGFLDESIVAL
AEM58055.1        DEFWSIPAEADDPAAGQWRDGPGAHFFETVRDR----LGELP--FFVEDLGFLDESIVAL
AHB66799.1        DEFWSIPAEADDPAAGQWRDGPGAHFFETVRDR----LGELP--FFVEDLGFLDESIVAL
AJF25101.1        DEFWSIPAEADDPAAGQWRDGPGAHFFETVRDR----LGELP--FFVEDLGFLDESIVAL
AAV46825.1        DEFWSIPAEADDPAAGQWRDGPGAHFFETVRDR----LGELP--FFVEDLGFLDESIVAL
QCP91534.1        DEFWSIPAEADDPAAGQWRDGPGAHFFETVRDR----LGELP--FFVEDLGFLDESIVAL
QUJ72368.1        DEFWSIPAEADDPAAGQWRDGPGAHFFETVRDR----LGELP--FFVEDLGFLDESIVAL
UBZ54001.1        DSYYAIPIDADNPAAGEWHSVPGYDFFETVREH----IGSLP--FIIEDLGFIDQALHDL
CCC40696.1        DAYYAIPVDADTPAAGEWQSVPGYDFFETIREH----IGSLP--FVVEDLGFIDQALHDL
CAJ52690.1        DAYYAIPVDSDTPAAGEWHSVPGYDFFETIREH----IGSLP--FVVEDLGFIDQALHDL
QCJ48434.1        DEYWAIPADADDPAAGSWRDAPGYDFFETIERE----FGDLP--FVVEDLGFIDEQLVAF
AHZ21176.1        DEYWAIDASSDNPEDGEWRDAPGHDFFETVERE----LGELP--FIVEDLGFLDASLVDL
AFK19479.1        DEYWAIDASSDNPEDGEWRDAPGHDFFETVERE----LGELP--FIVEDLGFLDASLVDL
AFK19479.2        DEYWAIDASSDNPEDGEWRDAPGHDFFETVERE----LGELP--FIVEDLGFLDASLVDL
QCQ75940.1        DEYWAIDASSDNPEDGEWRDAPGHDFFETVERE----LGELP--FIVEDLGFLDASLVDL
AKU07735.1        DEFWAIDADSDDPGNGEWRDAPGHDFFETVERE----LGDLP--FIVEDLGFLDASLVDL
QOS11852.1        DEFWAIDADSDDPGNGEWRDAPGHDFFETVERE----LGDLP--FIVEDLGFLDASLVDL
QIB78738.1        DEFWAIDADSDDPGDGEWRDAPGHDFFETVERE----LGDLP--FIVEDLGFLDASLVDL
ADE05003.1        DEFWAIDADSDDPGDGEWRDAPGHDFFETVERE----LDDLP--FVVEDLGFLDASLVDL
ADE05003.2        DEFWAIDADSDDPGDGEWRDAPGHDFFETVERE----LDDLP--FVVEDLGFLDASLVDL
QCC48543.1        DEFWAIPADSDSPADGEWRDAPGADFFETVGER----LGELP--FVVEDLGFLDQSLVDL
ADQ67101.1        DEYWAIPADAHSPAAGEWRDAPGHDFFETVEDQ----FGDLP--FIVEDLGFLDQRMTDL
QIQ76396.1        DEYWAIPADAHSPAAGEWRDAPGHDFFETVEDQ----FGDLP--FIVEDLGFLDQRMTDL
QIB74651.1        DEYWAIPADAHSPAAGEWRDAPGHDFFETVEDQ----FGDLP--FIVEDLGFLDQRMTDL
AIF04697.1        DSAWEIPAHHPTAKNGQWVQGPKAELLEAILEV----APAGK--IVAEDLGIIPPSVVEL
AIF05955.1        DSAWEIPAHHPTAKNGQWVQGPKAELLEAILEV----APAGK--IVAEDLGIIPPSVVEL
QEE14682.1        QAFWRISGTAKDAIDGKWIKAPGEDLFIEVQKQ----LGILP--IIAEDLGVITSEVEAL
ADL19304.1        AAYWEVPAGEKTAVNGRWARGPGEDLFRRVMRE----VPRLQ--LIAEDLGFITPDVVQL
AMD30778.1        AAYWEVPAGERTAVRGRWARGPGEDLFRAIMAA----APRLQ--LIAEDLGLITPDVVRL
AKT33963.1        VAYWEVPHGEETAVRGRWAPGPGEELFKAVEDA------LQR--LIAEDLGLITPDVVEL
ABL88479.1        VSYWEVPYGEPTAVRGRWAPGPGEELFKAVEET------LPR--LIAEDLGFITPDVVEL
ACB39188.1        AAYWEVPYGEPTAVRGRWAPGPGEELFRAAEDA------LPR--LIAEDLGFITPDVVEL
AEA11843.1        AAYWEVPAGEKTAVNGRWVPAPGGELLERARDE----LGGLK--IIAEDLGYITPDVVEL
ABO08194.1        AAYWEVPAGEPTAAGGRWVPGPGKRLFDAAAAE----VDLGR--LVAEDLGYITPDVEEL
ABP49821.1        VAYWEVPAGEKTAVNGRWVPAPGAELLEKARSE----LGELR--LIAEDLGYITPDVVEL
AFA40300.1        AAYWEVPAGEKTAVRGRWVPAPGAELLEKARAE----LGELR--LIAEDLGYITPDVVEL
AAL63326.1        VAYWEVPGGETTAVRGRWAPSPGRRLFEAAAAA----VDLGR--LIAEDLGYITPEVEAL
NP_559144.1       VAYWEVPGGETTAVRGRWAPSPGRRLFEAAAAA----VDLGR--LIAEDLGYITPEVEAL
AET33957.1        LAYWEIPGGETTAARGRWAPSPGKRLFDAASAA----VELGR--LIAEDLGYITPDVEAL
ABD40312.1        VGFWEIRAGSDTAIEGRWIEAPAWDFIRTLVKE----FPCLP--IIAEDLGIITSDVREI
QXO95450.1        VGYWEIKAGSDTAIDGRWISAPVWDLLRTWTKK----FPCFP--VIAEDLGIITPDVREV
QVV87979.1        VGYWEIKAGSDTAIDGRWISAPVWDLLRTWTKK----FPCFP--VIAEDLGIITPDVREV
CCJ37196.1        VQYWEVPVDEMTAENGRWVDAPGRDLFTLLARS----RSCLP--IIAEDLGYITSDVHEM
SAI88308.1        VQYWEVPAGETTAENGRWVDAPGRDFFTLLARS----RSCLP--IIAEDLGYITSDVHEM
CVK34217.1        VQYWEVPAGETTAENGRWVDAPGRDFFTLLARS----RSCLP--IIAEDLGYITSDVHEM
BBL68280.1        VQYWEVPAGDETARNGRWVDAPGRDLFTLLARS----RPCLP--IIAEDLGYITPDVHEM
ABN57193.1        VQFWEVPAGEETARNGHWVDAPGRDLFTLLARS----RSCLP--IIAEDLGHITPDVHEM
SAI88240.1        ADYYEVPAGDATAEHGTWVDGPGPGFFGVLARQ----FPCFA--IVAEDLGANTPAVQGL
```

```
CVK34142.1          ADYYEVPAGDATAEHGTWVDGPGPGFFGVLARQ----FPCFA--IVAEDLGANTPAVQGL
CCJ37132.1          ADYYEVPAGDATAEHGAWVDGPGPEFFEVLARQ----FPCFA--IVAEDLGANTPAVQGL
BBL68006.1          ADYYEIPAGDPTAEHGTWVDGPGAGFFEALVRK----HPCFA--IVAEDLGANTPAVQAL
ABN56987.1          ADYYEVPAGDATAERGTWVDGPGEDFFEALARK----HPCFP--IVAEDLGANTPAVQAL
                       : :         * *   *   .:                  . *::*        .

AUV80673.1          RTRYGLASLKLLPFVDWC-EEDHPHKPHRYTADTVAYTSTHDSETARGAYES-LSDEQRA
QCC51341.1          RDTLGYPGMVVAAFADWC-DGDSRYHPANYDANSVAYTSTHDTDTVVGWYDA-LDEQDRE
QSG15659.1          REDLGYPGMNVAAFTDWC-DGDSRYHPSSYDENSVAYTSTHDTDTVAGWYAD-LDEQQKD
QSG11910.1          RDDLGYPGMNVAAFADWC-DGDSRYHPVTYDENSVAYTSTHDTDTLVGWYED-LDDRQLD
QSG08987.1          RDDLGYPGMNVAAFADWC-DGDSRYHPVTYDENSVAYTSTHDTDTLVGWYED-LDDRQLD
QSG05483.1          RDDLGYPGMNVAAFADWC-DGDSRYHPVTYDENSVAYTSTHDTDTLVGWYED-LDDRQLD
QGN07616.1          RDAFEFPGMSVAAMADWC-DPSQGHHPASYTENTVAYASTHDTDTVPGWYAS-LSDAQRE
QSG11921.1          RDAFGFPGMRVAGMADWC-DDDHTHHPANYDDDSVAYASTHDTSTTPGWYED-LSETQKQ
QSG08998.1          RDAFGFPGMRVAGMADWC-DDDHTHHPANYDDDSVAYASTHDTSTTPGWYED-LSETQKQ
QSG05496.1          RDAFGFPGMRVAGMADWC-DDDHTHHPANYDDDSVAYASTHDTSTTPGWYED-LSETQKQ
CCQ33055.1          RDAFGFPGMRVAGMADWC-DAESTHHPASYDEPSVAYTSTHDTSTTVGWVKD-LSKKQRE
QSG02731.1          RRDIDAPGMKVMQYADWC-SEDHIYLPHTYEEDTVAYPGTHDTNTVRGWYEA-LEDEQVD
AUX09035.1          RRELGAPGMNVMQYADWC-TEDHGYQPHTYAADSVAYPSTHDTDTVCGYYEA-LGEDQRD
QFU81686.1          RQSIGAPGMRVLQYADWC-TEDHRYQPHVYPEDSVAYPATHDTNTVVGWYES-VDGEQRD
AWB27107.1          RDRFEFPGMRVPHYADWC-AERHRHKPAGYPERSVAYTSTHDTNTARGYVES-LDDRQRS
QLC34969.1          RDHLGFPGMRVPEYADWC-AEHHRYKPSDYPRDCVAYTSTHDTDTAVGYYES-LGERQRD
ACV11232.1          RDRFEFPGMRVPQYADWC-REGHRYKPTVYPDHCVGYTSTHDTDTAVGFYEK-LSAEQRD
QPV62520.1          RDRFEFPGMRVPHYADWC-AETDRYKPATYPRQSVGYTSTHDTDTAVGWYES-LSEEQRD
QLH84085.1          RDRFEFPGMRVPHYADWC-AETDRYKPATYPRQSVGYTSTHDTDTAVGWYES-LSAEQRD
QLH79093.1          RDRLEFPGMRVPHYADWC-AETDRYKPATYPRQSVGYTSTHDTDTAVGWYES-LSAEQRD
QAU11732.1          MAEFGFPGMRVPQYADWC-AEGNEYQPMHYSEGVVGYTSTHDTDTWAGYFDD-LPAEQRD
QAY20111.1          MAEFGFPGMRVPQYADWC-AEGNEYQPMHYGEGVVGYTSTHDTDTWAGYFED-LPAEQRD
QWC20591.1          MAEFGFPGMRVPQYADWC-AEGNEYQPMHYGEDVVGYTSTHDTDTWVGYFGD-LPAEQRD
QUO46499.1          MAEFGFPGMRVPQYADWC-AEGNEYQPIHYGEGVVGYTSTHDTDTWAGYFED-LPAAQRD
QKG93724.1          MAEFGFPGMRVPQYADWC-AEGNQYQPMHYGEGVVGYTSTHDTDTWVGYFED-LPPEQRD
QKY17977.1          MAEFGFPGMRVPQYADWC-QEGNPYQPMHYPEGVVGYTSTHDTDTWVGYFED-LPERQRD
ACM57093.1          MVEFGFPGMRVPQYADWC-AEGNQYQPMHYPEGVVGYTSTHDTDTWVGYFEG-LPERQRD
AZQ14643.1          MAEFGFPGMRVPQYADWC-AEGDQYQPMHYPEGVVGYTATHDTNTWVGYFES-LPERQRD
ATW89261.1          MDRFDFPGMRVPQYADWC-QGGNEHQPMNYPKNSVGYTSTHDTDTFDGYYQT-LSERQRD
ABQ75827.1          RESLNFPGMRVPQYADWC-QEGDMYQPMHYPRQCVAYTATHDTNTTVGYYNS-LSARQRD
QZX99028.1          REAFDLPGMRVPQYADWC-QQGHMYQPMHYPEDCVAYTSTHDTDTAVGYYES-LDDRQRD
QZP38174.1          REAFDLPGMRVPQYADWC-QQGHMYQPMHYPEDCVAYTSTHDTDTAVGYYES-LGDRQRD
QZY03180.1          REAFDLPGMRVPQYADWC-QQGHMYQPMHYPEDCVAYTSTHDTDTAVGYYES-LGDRQRD
AUG48211.1          RDQFGFPGMRVPQYADWC-EEGHMYQPMHYPESAVGYTSTHDTDTIVGYYRG-LSDRQRD
AEM58055.1          RDRFGFPGMRVPQYADWC-EEGHMYQPMHYPESAVGYTSTHDTDTIVGYYRD-LADRQRD
AHB66799.1          RDRFGFPGMRVPQYADWC-EEGHMYQPMHYPESAVGYTSTHDTDTIVGYYRD-LADRQRD
AJF25101.1          RDRFGFPGMRVPQYADWC-EEGHMYQPMHYPESAVGYTSTHDTDTIVGYYRD-LADRQRD
AAV46825.1          RDEFGFPGMRVPQYADWC-EEGHMYQPMHYPESAVGYTSTHDTDTIVGYYRD-LADRQRD
QCP91534.1          RDEFGFPGMRVPQYADWC-EEGHMYQPMHYPESAVGYTSTHDTDTIVGYYRD-LADRQRD
QUJ72368.1          RDEFGFPGMRVPQYADWC-EEGHMYQPMHYPESAVGYTSTHDTDTIVGYYRD-LADRQRD
UBZ54001.1          REYFDFPGMRVPHYADWC-REGDMYQPMHYPPQSVAYSSTHDTDTIVGYYDS-LSQSQRD
CCC40696.1          REHFNFPGMRVPHYADWC-HEGNMYQPMHYPAQSVAYSSTHDTNTIVGYYDS-LSHSQRD
CAJ52690.1          REHFNFPGMRVPHYADWC-HEGNMYQPMHYPAQSVAYSSTHDTNTIVGYYGS-LSHSQRD
QCJ48434.1          RDHFEFPGMRVPHYADWC-REGDMNQPMHYPHGSIGYTATHDTDTTVGYYRS-LPDDQTD
AHZ21176.1          RDRFAFPGMRVPQYADWC-REGDMYQPMHYPENSVAYTSTHDTDTFVGYYQS-LDDHTRD
AFK19479.1          RDRFAFPGMRVPQYADWC-REGDMYQPMHYPENSVAYTSTHDTDTFVGYYQS-LDDHTRD
AFK19479.2          RDRFAFPGMRVPQYADWC-REGDMYQPMHYPENSVAYTSTHDTDTFVGYYQS-LDDHTRD
QCQ75940.1          RDRFAFPGMRVPQYADWC-REGDMYQPMHYPENSVAYTSTHDTDTFVGYYQS-LDDHTRD
AKU07735.1          RDRFAFPGMRVPQYADWC-SEGDMYQPMHYPENSVAYTSTHDTDTFVGYYRD-LDDRTRD
QOS11852.1          RDRFAFPGMRVPQYADWC-SEGDMYQPMHYPENSVAYTSTHDTDTFVGYYRD-LDDRTRD
QIB78738.1          RDRFAFPGMRVPQYADWC-REGDMYQPMHYPESSVAYTSTHDTDTFVGYYRD-LDDRTRD
ADE05003.1          RDRFAFPGMRVPQYADWC-REGDMYQPMHYPESSVAYTSTHDTDTFVGYYRD-LDDRTRD
ADE05003.2          RDRFAFPGMRVPQYADWC-REGDMYQPMHYPESSVAYTSTHDTDTFVGYYRD-LDDRTRD
QCC48543.1          RERFGFPSMRVPHYADWC-QEGDMYQPMHYPENSVAYSSTHDTNTLVGYCEN-LSDRQRS
ADQ67101.1          RDRFGFPGMRVPQYANWC-QEGDMYQPMHFPENSVGYTSTHDTNTFVGYYRD-LPADQKD
QIQ76396.1          RDRFGFPGMRVPQYANWC-QEGDMYQPMHFPENSVGYTSTHDTNTFVGYYRD-LPADQKD
QIB74651.1          RDRFGFPGMRVPQYANWC-QEGDMYQPMHFPENSVGYTSTHDTNTFVGYYRD-LPADQKD
AIF04697.1          RKQNGIPGMAVLHFSND--DVDNPHNPENHSEDTVVYTGTHDNDTTVGWGGKP-------
AIF05955.1          RKQNGIPGMAVLHFSND--DVDNPHNPENHSEDTVVYTGTHDNDTTVGWGGKP-------
QEE14682.1          RDKFNFPGMKILQFAFGS-DEENPYLPHNFVHNCISYTGTHDNNTTLGWWQNDATKTEKK
ADL19304.1          RDRLGLPGLKVLQFAWDG-NPGNPYKPHNYGRNFVVYTGTHDNNTIVGWFFHEASPRARR
AMD30778.1          RERLGIPGLKVLQFAWDG-SPNNPYLPHNYARNFIVYTGTHDNNTIVGWFFHEASPRARA
AKT33963.1          RRRLGIPGMRVLQFAWDG-NPANEHKPHNYERNLVAYTGTHDNNTVVGWWRDEAPPRARR
ABL88479.1          RRRLGIPGMRVLQFAWDG-NPANEHKPHNYERNLVAYTGTHDNNTIVGWWREETTPRSRR
ACB39188.1          RYRLGIPGMRVLQFAWDG-NPANEHKPHNYERNLVAYTGTHDNNTTLGWWREETTPRSRR
AEA11843.1          RDRFGLPGMRVLQFAWDG-NPANEHKPHNHVKNSVVYTGTHDNNTIVGWFFREASPKARR
ABO08194.1          RDSLGLPGMRVLQFAWDG-NPANLHKPHNHVKNSLVYTGTHDNNTAVGWFTEEATPAVRR
ABP49821.1          RDRLGFPGMRVLQFAWDG-NPANEHKPHNHVKNSVVYTGTHDNNTAVGWYLEEATPRARR
AFA40300.1          RDRFGLPGMRVLQFAWDG-NPANEHKPHNHVKNSVVYTGTHDNNMAVGWYQEEATPRARR
AAL63326.1          REELGLPGMRVLQFAWDG-NPANPHKPHNHVKNAVVYTGTHDNNTAVGWYKEEATARARR
NP_559144.1         REELGLPGMRVLQFAWDG-NPANPHKPHNHVKNAVVYTGTHDNNTAVGWYKEEATARARR
AET33957.1          RDELGLPGMRVLQFAWDG-NPANPHKPHNHVKNSVVYTGTHDNNTAVGWYLQEASPRARR
```

```
ABD40312.1       MREFMIPGMKVLVFAFSSPTGDNPYILHNIPKDSVVYTGTHDNTPVRGWYELDATHEEKQ
QXO95450.1       MREFDIPGMKVLIFAFSSETGDNPYILHNIPPNTVVYTGTHDNTPVLGWFLHEATPEEKQ
QVV87979.1       MREFDIPGMKVLIFAFSSETGDNPYILHNIPPNTVVYTGTHDNTPVLGWFLHEATPEEKQ
CCJ37196.1       MAHFGFPGMKVLIFGFSGDVARNPHAPHNITGGCIAYTGTHDNNTVRGWFEHEVSGDQRD
SAI88308.1       MAHFGFPGMKVLIFGFSGDVARNPHAPHNITGGCIAYTGTHDNNTVRGWFEHEVSGEQRD
CVK34217.1       MAHFGFPGMKVLIFGFSGDVARNPHAPHNITGGCIAYTGTHDNNTVRGWFEHEVSGEQRD
BBL68280.1       MAYFGFPGMKILIFGFSGDVAQNPHAPHNFAKGYVAYTGTHDNNTVRGWFEHETSEEQKN
ABN57193.1       MAYFGFPGMKILTFGFSGDVAQNPHAPHNITEGSVAYTGTHDNNTVRGWFGREVPDDQKN
SAI88240.1       LDRFGFPGMKILLFAFGEGLPESAHIPHNYVPNLICYTGTHDNNTARGWFEEEASEEDKQ
CVK34142.1       LDRFGFPGMKILLFAFGEGLPESAHIPHNYVPNLICYTGTHDNNTARGWFEEEASEEDKQ
CCJ37132.1       LDRFGFPGMKILLFAFGEGLPGSAHIPHNYVPNLICYTGTHDNNTARGWFEEEASEEDKQ
BBL68006.1       LDRFDFPGMKILLFAFGEGIAGSAHIPHNYVHNLICYTGTHDNNTARGWFEEEATDEDKE
ABN56987.1       LDRFDFPGMKILLFAFGEGIARTPHIPHNYVHNLICYTGTHDNNTARGWFEEEASEEDKE
                        .: :                       : *  .***.    *

AUV80673.1       CFEAYFDGPVAAETVHETCIEAVWGSEANLALAQIQDVLGRGNEARFNVPGTTAGNWAWR
QCC51341.1       CLHYAV-DHEGGD-VHWDILDTVWNSDSVIALAQVQDPLGYGSEARFNVPGTVEGNWAWR
QSG15659.1       CLHYAV-DYEGGD-VHWDILDTIWGSDSVITLAQVQDPLGYGSDARFNVPGTEEDNWAWR
QSG11910.1       CLHYAV-DYEGGD-VHWDILDTVWGSDSAITLAQVQDPLGYGSEARFNVPGTEEGNWSWR
QSG08987.1       CLHYAV-DYEGGD-VHWDILDTVWGSDSAITLAQVQDPLGYGSEARFNVPGTEEGNWSWR
QSG05483.1       CLHYAV-DYEGGD-VHWDILDTVWGSDSVITLAQVQDPLGYGSEARFNVPGTEKGNWSWR
QGN07616.1       CLHDVV-DGNSDD-PHWNIVETIWASEAVLTLVQCQDLLGLGTAHRFNFPGTAEGNWEWR
QSG11921.1       CLHSVV-DHEGDE-VHWDIIEAVWASPAIITLAQFQDFLGLGDEHRFNLPGTATGNWKWR
QSG08998.1       CLHSVV-DHEGDE-VHWDIIEAVWASPAIITLAQFQDFLGLGDEHRFNLPGTATGNWKWR
QSG05496.1       CLHSVV-DHEGGE-VHWDIIEAVWASPAIITIAQFQDFLGLGDEHRFNLPGTATGNWKWR
CCQ33055.1       CLYFAV-DHEGGP-INWDVIETVWETPAVITFAQFQDFHGLGDEHRFNLPGTATGNWKWR
QSG02731.1       CLHYYL-GSDGSE-IHWDMIERAWHTASSLAVVPLQDLYGLGAEARFNTPGTESGNWDWR
AUX09035.1       CLHYYM-GTDGSE-IHWEFIEAAWGSDSVFAVAPLPDLFGLGSEARFNTPGTADGNWEWR
QFU81686.1       CLHYYL-STDGHE-IHWEFLEAAWESDAVFAITPLPDLYGLDGEARFNTPGTADGNWDWR
AWB27107.1       CLEYAL-ATDAE-RAVWDLLEAVWQSDAALAIAPVQDLLDLGSESRLNDPAGG-DDWTWR
QLC34969.1       CFDYAL-DFDGDESPAWALLDAVWDSPAVVAMTTLPDLLTLGSDARLNTPGTGTGNWAWR
ACV11232.1       CLEYAL-ATDGD-SIAWDLIEAVWHSDAALAMTTVPDLLERGSDARLNQPGTGEGNWTWR
QPV62520.1       CLHYAL-ATDGE-RVAWDLIEAVWDSDAALALTTVPDLLELGSEARFNVPGTAEGNWRWR
QLH84085.1       CLHYAL-ATDGE-RIAWDLIEAVWDSDAALALTTVPDLLELGSEARFNVPGTAENNWRWR
QLH79093.1       CLHYAL-ATDGE-RIAWDLIEAVWDSDAALALTTVPDLLELGSEARFNVPGTAEGNWRWR
QAU11732.1       CFRYNV-GAGPDEPVEWAIVDEVWASDAILAVTTLQDLLGLGSEARFNEPGTLAGNWEWR
QAY20111.1       CFRYNV-GSDPDEPPEWAIIDEVWSSDAILAVATIQDLLGLGSESRFNEPGTLDGNWEWR
QWC20591.1       CFRYNV-GSDPDEPTEWAIIDEVWSSDAILAVTTVQDLFGLGSEARFNEPGTLAGNWEWR
QUO46499.1       CFRYNV-GSDPDEPSEWAIIDAVWGSDAILAVTTLQDLLGLGSEARFNEPGTLDGNWEWR
QKG93724.1       CFRYNV-GSDPEEPPEWAIIDAVWGSDAILAVTTLQDLLGLGSDARFNEPGTLAGNWEWR
QKY17977.1       CFRYNV-GADGDREVEWEIIDEVWGSEAVLAMTTVQDLLGLGSEARFNEPGTIDGNWEWR
ACM57093.1       CFRYNV-GADGDRPPEWEIIDEVWASEAVLAMTTVQDLLGLGSESRFNEPGTLNGNWEWR
AZQ14643.1       CFRFNV-GADGDRAPEWEIIDEVWASGAVLAMTTLPDLLGRGSEARFNEPGTLDGNWEWR
ATW89261.1       CLQYNL-GVDGSE-INWSMIEAVWRSDAVLAFTTMQDLLGLDSHARFNTPGTLDGNWTWR
ABQ75827.1       CLHYNL-GIDGHE-INWSLIDAVWRSDAIIAMTTMQDILGLDSHARLNTPGTAVGNWQWR
QZX99028.1       CLHYNL-GTDGVD-IEWDLVEAVWNSEATLAVTTVQDLLGLDSHARFNTPGTTDGNWRWR
QZP38174.1       CLHYNL-GTDGVE-IEWDLVEAVWNSEATLAVTTMQDLLGLDSHARFNTPGTTEGNWRWR
QZY03180.1       CLHYNL-GTDGVE-IEWDLVEAVWNSDATLAVTTMQDLLGLDSHARFNTPGTTEGNWRWR
AUG48211.1       CLHYNL-GTDGDE-INWDLIEAVWNSNAVVAMTTMQDLLGLGSDARFNVPGTATGNWQWR
AEM58055.1       CLHYNL-GTDGDE-INWDLIEAVWNSNAVVAMTTMQDLLGLDSDARFNVPGTATGNWQWR
AHB66799.1       CLHYNL-GTDGDE-INWDLIEAVWNSNAVVAMTTMQDLLGLDSDARFNVPGTATGNWQWR
AJF25101.1       CLHYNL-GTDGDE-INWDLIEAVWNSNAVVAMTTMQDLLGLDSDARFNVPGTATGNWQWR
AAV46825.1       CLHYNL-GTDGDE-INWDLIEAVWNSNAIVAMTTMQDLLGLGSDTRFNVPGTATGNWQWR
QCP91534.1       CLHYNL-GTDGDE-INWDLIEAVWNSNAIVAMTTMQDLLGLGSDTRFNVPGTATGNWQWR
QUJ72368.1       CLHYNL-GTDGDE-INWDLIEAVWNSNAIVAMTTMQDLLGLGSDTRFNVPGTATGNWQWR
UBZ54001.1       CLHYNL-GVDGSE-INWSIIDAVWRSDAKIGLTTLQDVLGLDSHARFNEPGTASGNWEWR
CCC40696.1       CLHYNL-GVDGSE-INWSIIDAVWRSDAQIGFTTLQDILGLDSHARFNEPGTASGNWQWR
CAJ52690.1       CLHYNL-GVDGSE-INWSIIDAVWRSDAQIGFTTLQDILGLDSHARFNEPGTASGNWQWR
QCJ48434.1       CLHYNL-GVDGSD-INWSLIEAVWNSEAVLAMTTVPDLLGLGSEARFNTPGTAEGNWSWR
AHZ21176.1       CLHYNI-GADGSE-IHWSIIDAVWNSASVLAFTTVQDLLGLDSRARFNTPGTQNGNWEWR
AFK19479.1       CLHYNI-GADGSE-IHWSIIDAVWNSASVLAFTTVQDLLGLDSRARFNTPGTQNGNWEWR
AFK19479.2       CLHYNI-GADGSE-IHWSIIDAVWNSASVLAFTTVQDLLGLDSRARFNTPGTQNGNWEWR
QCQ75940.1       CLHYNI-GADGSE-IHWSIIDAVWNSASVLAFTTVQDLLGLDSRARFNTPGTQNGNWEWR
AKU07735.1       CLHYNI-GADGSE-IHWSIIDAVWNSASVLAFTTTQDLLGLGSDARFNTPGTQTGNWEWR
QOS11852.1       CLHYNI-GADGSE-IHWSIIDAVWNSASVLAFTTTQDLLGLGSDARFNTPGTQTGNWEWR
QIB78738.1       CLHYNI-GADGSE-IHWSIIDAVWNSASVLAFTTTQDLLGLGSDARFNTPGTQTGNWEWR
ADE05003.1       CLHYNI-GADGSE-IHWSIIDAVWNSASVLAFTTTQDLLGLGSDARFNTPGTQTGNWEWR
ADE05003.2       CLHYNI-GADGSE-IHWSIIDAVWNSASVLAFTTTQDLLGLGSDARFNTPGTQTGNWEWR
QCC48543.1       CLEYNI-GADGSE-LHWSMIEAVWRADSVLAFTTMQDVLGLDEHARFNRPGTTSGNWSWR
ADQ67101.1       CLHYNI-GADGSE-IHWSIIDAVWRSNAVIAFTTIQDILGLGAEARFNVPGTTDGNWRWR
QIQ76396.1       CLHYNI-GADGSE-IHWSIIDAVWRSNAVIAFTTIQDILGLGAEARFNVPGTTDGNWRWR
QIB74651.1       CLHYNI-GADGSE-IHWSIIDAVWRSNAVIAFTTIQDILGLGAEARFNVPGTTDGNWRWR
AIF04697.1       ---------------VQDLIQDALNSPANLAIIPLQDVMGLDSQARMNTPGTLDGNWEWG
AIF05955.1       ---------------VQDLIQDALNSPANLAIIPLQDVMGLDSQARMNTPGTLDGNWEWR
QEE14682.1       NLREYLNTKSENKSVVDDLIRILYRSIAKVAIIPIQDLLGQGEEARMNTPSVEGGNWQYQ
ADL19304.1       EAMNYMGLR-DAREINWAFIRLAMMSVADVSIFPVQDVLGLGPEARMNTPGTVGNNWRWR
AMD30778.1       QALRYMGLN-SARDINWAFIRLAMMSVADVSIFPAQDVLGLGPEARMNTPGTVSNNWLWR
AKT33963.1       EALAYMGGC-R-GAVNWCFIRLLFSTVADVAVVPIQDVLGLGAEARINKPATSRGNWKWR
```

```
ABL88479.1       EALAYIGGC-G-REINWCFIRLLFSTVADVAVVPIQDVLGLGSEARINKPGTLRGNWKWK
ACB39188.1       EALAYMGGC-R-GGVSWCFIRLLFSTVADVAVVPMQDALGLGSEARMNKPGTARGNWKWR
AEA11843.1       EALEYMGRR-SAKDIHWAFIRLAYFSVADVAVVPMQDFMGLGPEARINRPGTRGGNWVWR
ABO08194.1       EVLKYGNCR-P-REINWCLIRLAHMSVADVAIVPMQDVLGLGSEARMNKPGTVGGNWRWR
ABP49821.1       EFCQYAKCS-AAEGVHWCFIRLAYMSVANVAIVPIQDVLGLGSEARMNKPGTVGGNWRWR
AFA40300.1       EFREYAKCA-SGEGVNWCFIRLAHMSVADVAIVPMQDVLGLGSEARMNKPGTMGGNWRWR
AAL63326.1       EFKKYSNCA---GEVHWCFIKLAHMSVANVAIVPVQDLLGLGSEARMNRPGTVGGNWKWR
NP_559144.1      EFKKYSNCA---GEVHWCFIKLAHMSVANVAIVPVQDLLGLGSEARMNRPGTVGGNWKWR
AET33957.1       EFRQYSNCR---EAVNWCFIKLAYMSVADVAVVPMQDVLGLGPEARMNRPGTVGGNWRWR
ABD40312.1       RLIAYLGREPEIHEISDIFIRLAMMSSANTAIIPIQDILGLDSSARMNKPGTEHGNWRWR
QXO95450.1       RFVSYIGYEVSSEKVSDVFVRLAMMSSANTAIISMQDVLGLDISARMNRPGSEKGNWRWM
QVV87979.1       RFVSYIGYEVSSEKVSDVFVRLAMMSSANTAIISMQDVLGLDISARMNRPGSEKGNWRWM
CCJ37196.1       LLFRYIGGTFGADHAHRILIRLAMLSPAETVIVPMQDILGLGEEARMNRPGTAEGNWEWR
SAI88308.1       LLFRYIGGSFGAEHVHRILIRLAMLSPAETVIVPMQDILGLGEEARMNRPGTAEGNWEWR
CVK34217.1       LLFRYIGGSFGAEHVHRILIRLAMLSPAETVIVPMQDILGLGEEARMNRPGTAEGNWEWR
BBL68280.1       LLFRYIGGSVSGDQVHRILIRLAMLSPADTVIVPMQDILGLGEEARMNRPGTTEANWEWR
ABN57193.1       LLFRYIGGPVSGDEIHRILIRLAMLSPADTVIVPMQDILGLGEEARMNRPGTTEGNWEWR
SAI88240.1       RFFAYIGREVTAEEVPRELIRLAMTSVARTSIIPMQDILGLGAAARMNYPSTSDGNWEWR
CVK34142.1       RFFAYIGREVTAEEVPRELIRLAMTSVARTSIIPMQDILGLGAEARMNYPSTSDGNWEWR
CCJ37132.1       RFFAYIGREVAADEVPRELIRLAMTSVARASIIPMQDILGLGAEARMNYPSTSDGNWEWR
BBL68006.1       RFFAYAGREVRADSVHREFVRLGMTSVARACIVPMQDVLGLGAAARMNYPSTTTGNWEWR
ABN56987.1       RFFTYIGREVAADEVHRELVRLAMTSVARACIVPMQDVLGLGAAARMNYPSTTTGNWVWR
                                    :     : :   .    *    .   *:* *.    :* :

AUV80673.1       VREEQLTD-AAAGWLAEATRDANR-------
QCC51341.1       VSEDALSG-DVVQNLYDITEESDRLAE----
QSG15659.1       VSEDALSA-DVAERLYEITESSGRLTD----
QSG11910.1       VSEDALTD-EVAQRLYDITEATGRLTD----
QSG08987.1       VSEDALTD-EVAQRLYDITEATGRLTD----
QSG05483.1       VGEDALTD-EVAQRLYDITEATGRLTD----
QGN07616.1       VLPSELTD-DVAERLAAVSRRTDRA------
QSG11921.1       MRRSELDP-AVAERLADVTRRTDRR------
QSG08998.1       MRRSELDP-AVAERLADVTRRTDRR------
QSG05496.1       MRRSELDP-AVAERLADVTRRTDRR------
CCQ33055.1       MLDSELDS-AVADRLADVTRKADRT------
QSG02731.1       CTDELL-ESFPTDRLRTITDEADR-------
AUX09035.1       CPAALITAEFPTERLRAVTDGTDRVTLN---
QFU81686.1       LSTDRL-ESFPTARLRELTARTDRLDETTSH
AWB27107.1       LPAGALDD-ALADRLRATTDAALR-------
QLC34969.1       VDEGALAD-DLAARLADVTANARR-------
ACV11232.1       VTADELDA-DTADRLAAVTRASLR-------
QPV62520.1       VTADQLDP-DVADELAGVTAATLR-------
QLH84085.1       VTADQLDP-AVAGELAGVTAATLR-------
QLH79093.1       VTADRLDP-AVADELAGVTAATLR-------
QAU11732.1       VTREALDD-AIADRLWELAGDHVR-------
QAY20111.1       VARDDLDD-AIADRLWELAGKHVR-------
QWC20591.1       VSRDALDD-AIADRLWELGGTHVR-------
QUO46499.1       VARDALDD-EIADRLWELSGKHVR-------
QKG93724.1       VARDALDD-EIADRLWELGGRHVR-------
QKY17977.1       VTRDALDD-AVADRLWDLAGRHVR-------
ACM57093.1       VRRDALDD-AIADRLWDLAGRHVR-------
AZQ14643.1       VRREALDD-DIADRLFELAGRHVR-------
ATW89261.1       VTSEGLDE-RIAKKLGTMTAIEIR-------
ABQ75827.1       CTSEGLSD-ALAGRLAALSAEHIRD------
QZX99028.1       VTEAGLDG-AVADRLARITDATVR-------
QZP38174.1       VTEDGLND-AVAERLARITDATVR-------
QZY03180.1       VTEDGLND-AVAERLARITDATVR-------
AUG48211.1       VTDAALDA-DVAERLRRVTDRTIR-------
AEM58055.1       VTEDAIND-EVAERLRQVTDRTIR-------
AHB66799.1       VTEDAIND-EVAERLRQVTDRTIR-------
AJF25101.1       VTEDAIND-EVAERLRQVTDRTIR-------
AAV46825.1       VTDTALDA-DIAERLRRVTDRTIR-------
QCP91534.1       VTDTALDA-DIAERLRRVTDRTIR-------
QUJ72368.1       VTEDALDT-DVAERLRQVTDRTIR-------
UBZ54001.1       CTDSGLDE-DLARRLAGLTLEHVRD------
CCC40696.1       CTDSGLDD-DLARRLAGLTIEHVRD------
CAJ52690.1       CTDSGLDD-DLARRLAGLTIEHVRD------
QCJ48434.1       CTYEGLDE-SVAERLRDVTDFTIR-------
AHZ21176.1       VTREGLDD-DVSARLAELTDLHIRN------
AFK19479.1       VTREGLDD-DVSARLAELTDLHIRN------
AFK19479.2       VTREGLDD-DVSARLAELTDLHIRN------
QCQ75940.1       VTREGLDD-DVSARLAELTDLHIRN------
AKU07735.1       VTREGLDD-GVADRLANLTDLHIRN------
QOS11852.1       VTREGLDD-GVADRLANLTDLHIRN------
QIB78738.1       VTREGLDD-GVADRLASLTDLHIRN------
ADE05003.1       VTREGLDD-GVADRLASLTDLHIRN------
ADE05003.2       VTREGLDD-GVADRLASLTDLHIRN------
QCC48543.1       CTVEAFSE-ALADRLARLTDEHIRN------
```

```
ADQ67101.1       CTEEGFDE-ELASRLARLTDEHIR-------
QIQ76396.1       CTEEGFDE-ELASRLARLTDEHIR-------
QIB74651.1       CTEEGFDE-ELASRLARLTDEHIR-------
AIF04697.1       FDWDELTN-DSLNWFGQQVYRSGRGN-----
AIF05955.1       FDWDELTN-DSLNWFGQQVYRSGRGN-----
QEE14682.1       VKIDNDVK-NRAKFLKKITKLYGRKVII---
ADL19304.1       LESFDGLR-RAARRLRDMTRAYGR-------
AMD30778.1       LDSLEGLW-RTASRLRDMTRSYGR-------
AKT33963.1       LHAQPP-P-TYARRLRRLAKLYGR-------
ABL88479.1       LAKDPP-I-NFAVRLRRLAKLYGR-------
ACB39188.1       MAGDPP-R-AVAARLRRLARIYGR-------
AEA11843.1       MTELPG-R-ALARRIRRLARLYGR-------
ABO08194.1       LAEQPP-R-ALLRKLREVTRLYGR-------
ABP49821.1       LAKMPN-A-AVRRRLRKLTRIYGR-------
AFA40300.1       LAKMPD-A-AVLRRLRKLTRTYGR-------
AAL63326.1       LGAQPE-P-RVWKRLRDVTRTYGR-------
NP_559144.1      LGAQPE-P-RVWKRLRDVTRTYGR-------
AET33957.1       MAKQPE-P-RQWRRLRSLARLYGR-------
ABD40312.1       LQERMISE-ELSRRLSELVKIYGR-------
QXO95450.1       MQERMITE-DLAYYIRTLTTTYGRL------
QVV87979.1       MQERMITE-DLAYYIRTLTTTYGRL------
CCJ37196.1       LRREQMRE-QAMQEFSEVTGLYGRK------
SAI88308.1       LRREQMRE-QAMQEFSEVTGLYGRK------
CVK34217.1       LRREQMRE-QAMQEFSEVTGLYGRK------
BBL68280.1       LRPDQIGE-ESMRELAEVTGIYGRG------
ABN57193.1       LRPDQAGE-ESMREFAEVTGIYGRG------
SAI88240.1       MTPAEFAG-APFDRLRRLTELCGRA------
CVK34142.1       MTPAEFAG-APFDRLRRLTELCGRA------
CCJ37132.1       MTPREFAD-APFDRLGRFTELCGRA------
BBL68006.1       MTPEEFAG-APFEWLRELTELTGRG------
ABN56987.1       MTPEEFAG-APFEWLRGLTELTGRG------
                               :        *
```

Figure S2. Clustal alignment of Hw-A and 83 archaeal amylomaltases present in the CAZy GH77 family. Hw-A is reported in red.

Figure S3

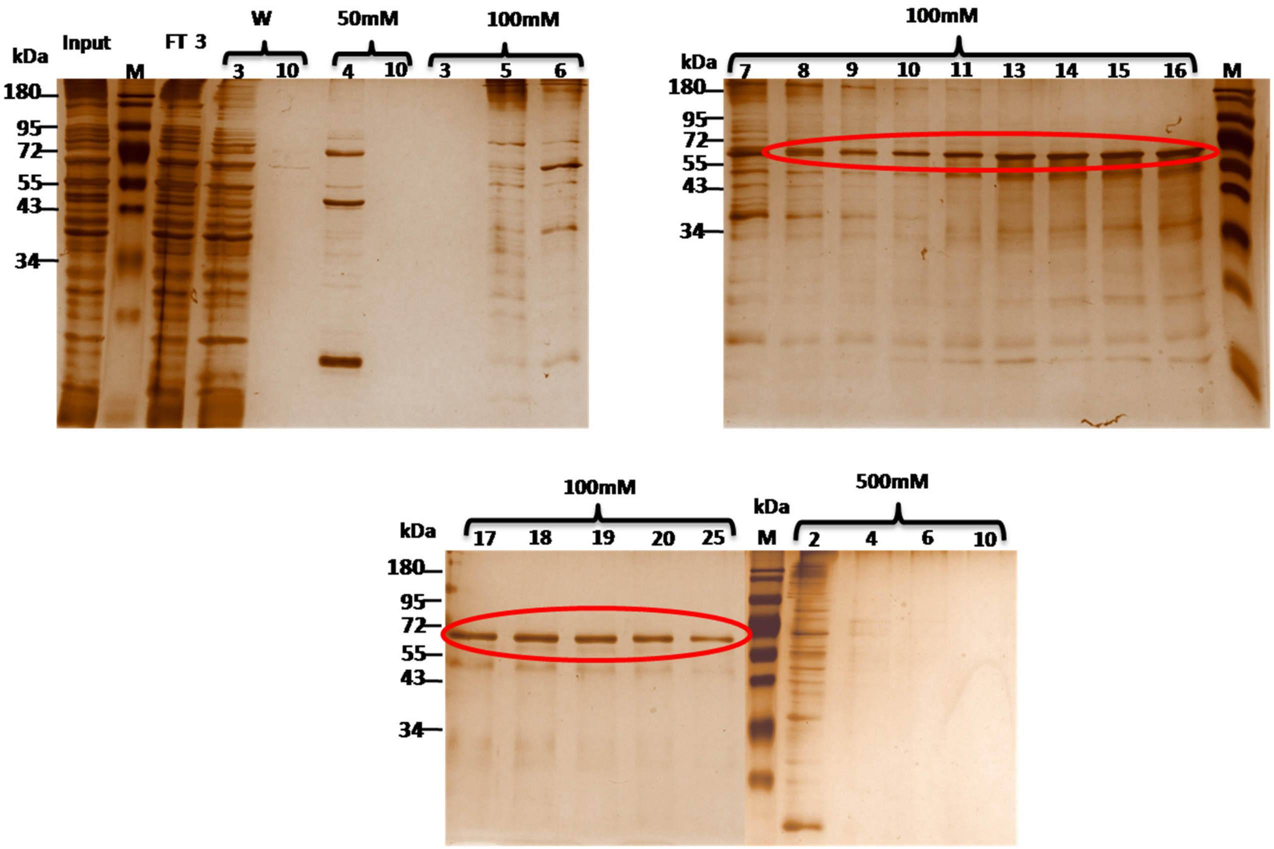

Figure S3.SDS-PAGE on 15% polyacrylamide gel of the fractions obtained from IMAC chromatography. Input: Soluble fraction of sonicated induced expression cells;M: Marker Thermo Scientific Page Ruler Prestained Protein Ladder; FT: flow-through; W: wash buffer; 50mM: fraction eluted with 50mM imidazole elution buffer; 100mM: fraction eluted with 100mM imidazole elution buffer; 500mM: fraction eluted with 500mM imidazole elution buffer.The recombinant protein was circled in red. Protein electrophoretic bands were revealed by silver staining.
